# One-step synthesis of imidazoles from Asmic (anisylsulfanylmethyl isocyanide)

**DOI:** 10.3762/bjoc.17.106

**Published:** 2021-06-24

**Authors:** Louis G Mueller, Allen Chao, Embarek AlWedi, Fraser F Fleming

**Affiliations:** 1 Department of Chemistry, Drexel University, 32 South 32nd St., Philadelphia PA 19104, USA; 2 Abzena, 360 George Patterson Blvd, Bristol, PA 19007, USA; 3 Merck Inc., 90 E. Scott Ave, Rahway, NJ 07065, USA

**Keywords:** Asmic, cyclization, imidazoles, isocyanides, nitriles

## Abstract

Substituted imidazoles are readily prepared by condensing the versatile isocyanide Asmic, anisylsulfanylmethylisocyanide, with nitrogenous π-electrophiles. Deprotonating Asmic with lithium hexamethyldisilazide effectively generates a potent nucleophile that efficiently intercepts nitrile and imine electrophiles to afford imidazoles. In situ cyclization to the imidazole is promoted by the conjugate acid, hexamethyldisilazane, which facilitates the requisite series of proton transfers. The rapid formation of imidazoles and the interchange of the anisylsulfanyl for hydrogen with Raney nickel make the method a valuable route to mono- and disubstituted imidazoles.

## Introduction

**Figure 1:**
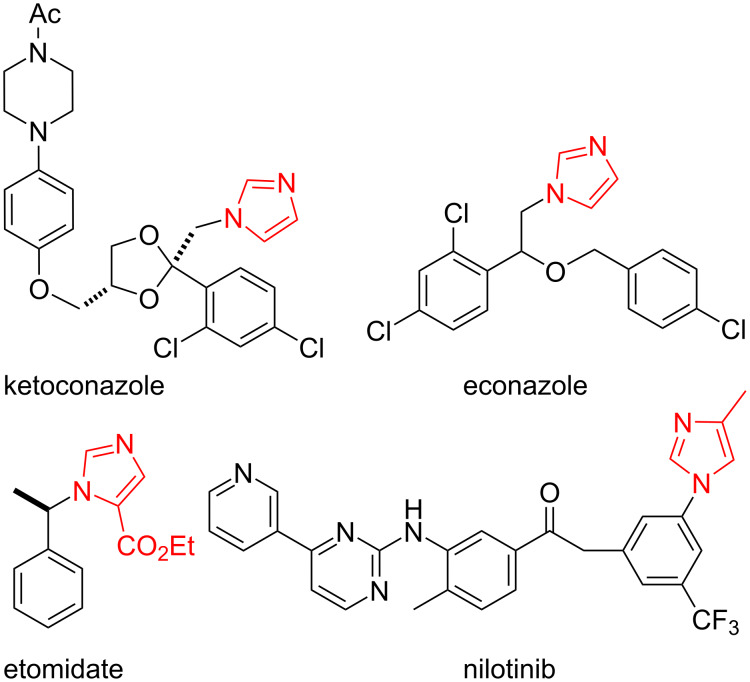
Representative imidazole-containing pharmaceuticals.

The imidazole core is the seventh most prevalent heterocycle among nitrogen-containing pharmaceuticals [1]. The privileged efficacy of imidazoles emanates from the central role of histidine in biological machinery, particularly as a base at enzymatic active sites [2]. As histidine mimics, imidazole-containing pharmaceuticals are often only *N*-substituted, as in the fungicides ketoconazole and econazole (Figure 1) [3], or disubstituted as illustrated by the anesthetic etomidate [4] and the antileukemia agent nilotinib [5].

**Scheme 1:**
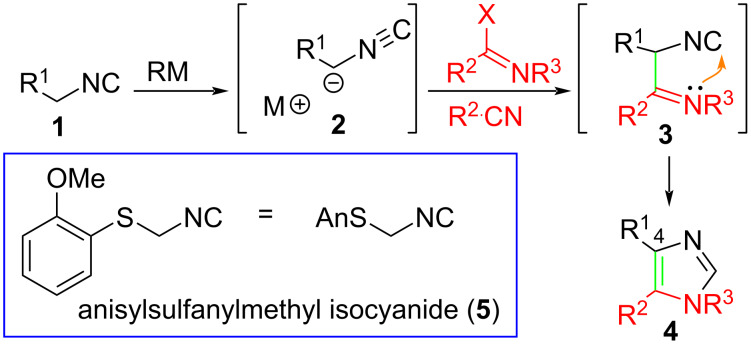
Asmic-condensation approach to imidazoles.

The outstanding and diverse bioactivity of imidazole-containing pharmaceuticals [6], as well as their role as ligands for transition metals [7], and organocatalysis [8], has stimulated an array of creative syntheses [9–10]. Among the numerous routes to imidazoles [11–12], the condensation of metalated isocyanides with nitrogenous π-electrophiles is distinguished by excellent efficiency and modularity. Deprotonating an isocyanide **1** affords an isocyanide-stabilized anion **2** whose condensation with an imidate or nitrile generates a transient imine **3** that readily cyclizes to afford imidazole **4** (Scheme 1). The excellent efficiency is somewhat countered by requiring isocyanides that are readily deprotonated; metalation of alkylisocyanides is challenging except for methyl isocyanide which is why most isocyanides employed in accessing imidazoles contain an adjacent electron withdrawing group (**1**, R^1^ = EWG) [13]. Installation of an electron withdrawing group adjacent to an isocyanide facilitates the deprotonation but creates weak nucleophiles **2** that are insufficiently nucleophilic to react with nitriles [14]. Described below is the use of Asmic, anisylsulfanylmethylisocyanide (**5**) [15], whose deprotonation affords a potent nucleophile that reacts directly with nitriles to provide an efficient, general approach to an array of imidazoles; Asmic is a crystalline, virtually odorless isocyanide with the advantage over related methods [16–17] in being readily prepared in fewer steps on at least 20 g scale [18], applicable for the synthesis of several heterocycles [19–20], and able to generate imidazoles from a broad array of nitrile and imidate electrophiles.

## Results and Discussion

Exploratory deprotonation of Asmic (**5**) with BuLi followed by addition of butyronitrile afforded an essentially quantitative conversion to imidazole **7a** (cf. Table 1, entry 1). An analogous reaction with benzonitrile gave a significantly lower yield of imidazole **7f** (cf. Table 1, entry 6) suggesting that the cleaner reaction profile with butyronitrile might have benefited from the acidic methylene protons of butyronitrile functioning as a proton shuttle during the cyclization cascade. Screening weaker bases with stronger conjugate acids to facilitate the requisite proton transfers identified LiHMDS as optimal; the LiHMDS-promoted condensation of Asmic with benzonitrile afforded imidazole **7f** in 93% yield (Table 1, entry 6). Presumably the cyclization of **6** is followed by protonation at the former isocyanide carbon by HMDS (the emerging C-2 of the imidazole) with the reformed LiHMDS deprotonating C-4 to form the imidazole ring.

**Table 1:** Asmic condensations to imidazoles.

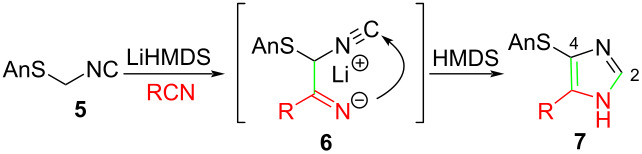			

Entry	Imidazole	Entry	Imidazole

1	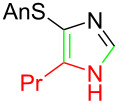 **7a**, (99%)	7	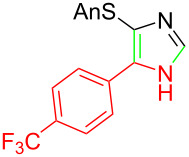 **7g**, (77%)
2	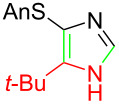 **7b**, (76%)	8	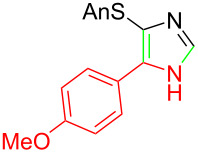 **7h**, (65%)
3	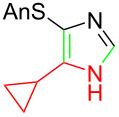 **7c**, (88%)	9	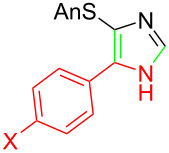 **7i**, X = F (60%) **7j**, X = F (80%) **7k**, X = F (61%)
4	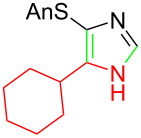 **7d**, (67%)	10	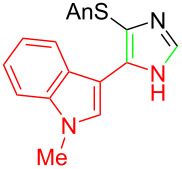 **7l**, (78%)
5	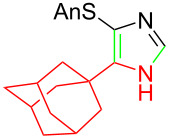 **7e**, (57%)		
6	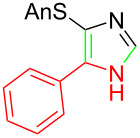 **7f**, (93%)		

^a^Prepared by trapping with methyl N-phenylformimidate.

**Scheme 2:**
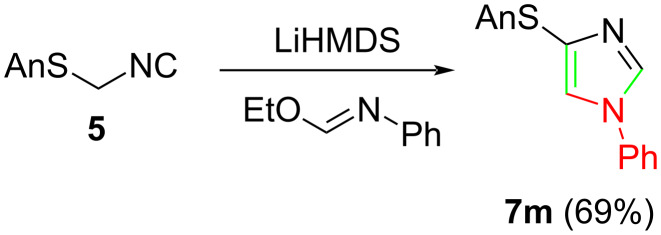
Asmic condensation with methyl *N*-phenylformimidate.

Identifying LiHMDS as the optimal base allowed the scope of the Asmic-based imidazole synthesis to be explored (Table 1). Aliphatic nitriles including cyclopropanecarbonitrile, cyclohexanecarbonitrile, and the sterically demanding adamantanecarbonitrile efficiently gave the corresponding imidazoles (**7a–e**) (Table 1, entries 1–5). Aryl nitriles with electron-donating or withdrawing substituents were competent reaction partners, providing a range of aryl-substituted imidazoles (Table 1, entries 6–10). Most reactions were complete in under one hour, though the less electrophilic *p*-methoxybenzonitrile required 2.5 h to provide imidazole **7h** (Table 1, entry 8). Trapping lithiated Asmic with 1-methyl-1*H*-indole-3-carbonitrile afforded indole **7l** whereas trapping with ethyl *N*-phenylformimidate afforded the selectively N-1 protected imidazole **7m** (Scheme 2) [21]. Collectively, the condensations of lithiated Asmic with nitriles or an imidate provides an efficient route to substituted imidazoles.

**Scheme 3:**
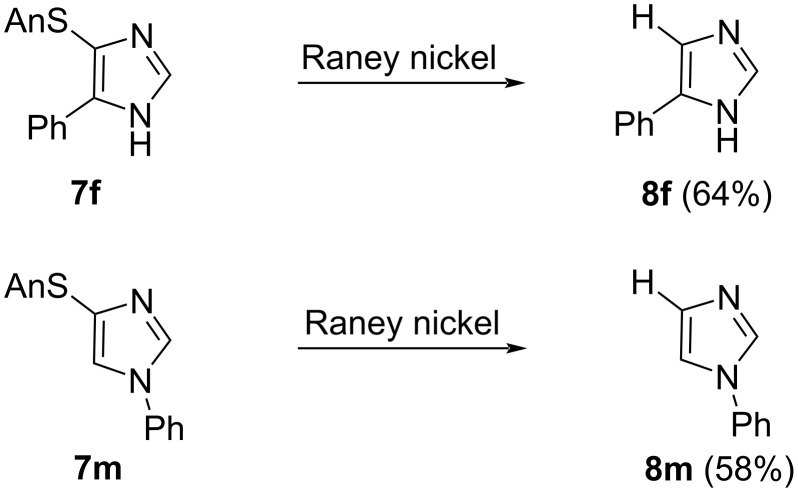
Anisylsulfanylimidazole reduction to monosubstituted imidazoles.

Raney nickel hydrogenolysis was effective in interchanging the C4 anisylsulfanyl group for hydrogen (Scheme 3); attempted lithium–anisylsulfanyl exchange [19] or palladium- [22] or nickel- [23] anisylsulfanyl cross coupling was not successful. Raney nickel reduction of **7f** and **7m** afforded the monosubsituted imidazoles **8f** and **8m**, respectively.

## Conclusion

Deprotonating Asmic with LiHMDS and trapping with nitriles or imidate electrophiles provides a robust, efficient synthesis of imidazoles. The method is rapid, modular and efficient. The anisylsulfanyl substituent serves as a valuable handle to the corresponding C-4 unsubstituted imidazoles providing an efficient route to diverse monosubstituted imidazoles.

## Supporting Information

File 1 Experimental procedures and spectral data of all synthesized compounds.

File 2 Raw FID files.
