# Supplementary material for: One-step synthesis of imidazoles from Asmic (anisylsulfanylmethyl isocyanide)
Source: Beilstein J Org Chem. 2021 Jun 24;17:1499–502. doi: 10.3762/bjoc.17.106 (PMC8239262; doi:10.3762/bjoc.17.106)
Supplement: File 1 — Experimental procedures and spectral data of all synthesized compounds. [file Beilstein_J_Org_Chem-17-1499-s001.pdf]

BEILSTEIN JOURNAL OF ORGANIC CHEMISTRY

Supporting Information

for

One-step synthesis of imidazoles from Asmic (anisylsulfanylmethyl isocyanide)

Louis G. Mueller, Allen Chao, Embarek AlWedi and Fraser F. Fleming

Beilstein J. Org. Chem. **2021,** *17,* 1499–1502. doi:10.3762/bjoc.17.106

Experimental procedures and spectral data of all synthesized compounds

License and Terms: This is a supporting information file under the terms of the Creative Commons Attribution License (https://creativecommons.org/licenses/by/4.0). Please note that the reuse, redistribution and reproduction in particular requires that the author(s) and source are credited and that individual graphics may be subject to special legal provisions.
The license is subject to the *Beilstein Journal of Organic Chemistry* terms and conditions: (https://www.beilstein-journals.org/bjoc/terms)

Compound	Procedure	^{1}H NMR	^{13}C NMR
General experimental procedures	S3		
General imidazole synthesis	S4		
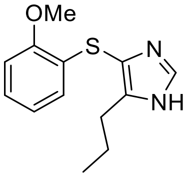 **7a**	S5	S12	S12
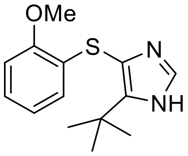 **7b**	S5	S13	S13
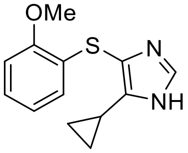 **7c**	S5	S14	S14
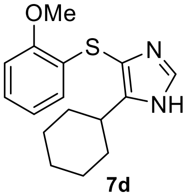 **7d**	S6	S15	S15
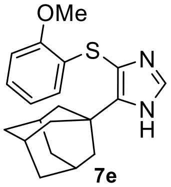 **7e**	S6	S16	S16
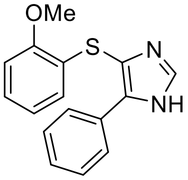 **7f**	S7	S17	S17
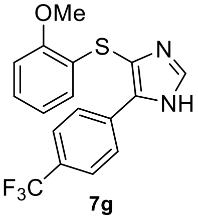 **7g**	S7	S18	S18

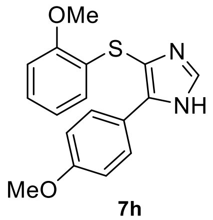 **7h**	S8	S19	S19
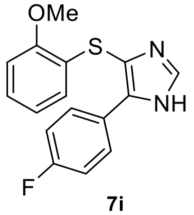 **7i**	S8	S20	S20
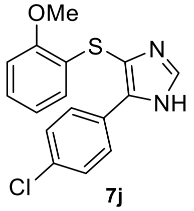 **7j**	S9	S21	S21
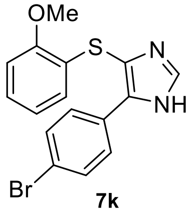 **7k**	S9	S22	S22
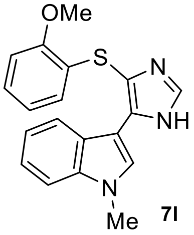 **7l**	S10	S23	S23
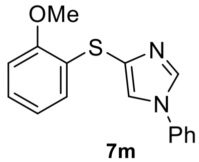 **7m**	S10	S24	S24
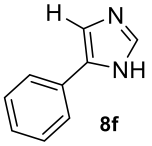 **8f**	S10	S25	S25
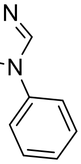 **8m**	S11	S26	S26

References	S27

General experimental procedures

All nonaqueous reactions were performed in flame-dried glassware under a nitrogen atmosphere. All chemicals were purchased from commercial vendors and used as received unless otherwise specified. Anhydrous tetrahydrofuran (THF) was distilled from benzophenone-sodium under N_2 before use. Reactions were magnetically stirred and monitored by thin layer chromatography (TLC) with 250 µm precoated silica gel plates or by mass spectrometry with an Advion S-CMS (ASAP Probe). Reactions requiring heating were heated in an oil bath. TLC plates were visualized using a UV lamp (254 nm). ^{1}H NMR and ^{13}C NMR high resolution nuclear magnetic resonance spectra were recorded on a Varian Mercury Plus 400 (400 MHz/101 MHz) or Varian Unity Inova 500 (500 MHz/126 MHz) spectrometers at room temperature. Chemical shifts are reported relative to $CDCl_3$ (δ 7.26) for ^{1}H NMR and TMS (δ 0.00) for ^{13}C NMR. Proton and ^{13}C and ^{1}H NMR spectra were obtained in suitable deuterated solvents. IR spectra were recorded as thin films (PerkinElmer Spectrum 100 FT-IR Spectrometer). High-resolution mass spectra were obtained on a Thermo-Electron LTQ-FT 7T Fourier transform ion cyclotron resonance (FT-ICR) spectrometer with an atmospheric pressure chemical ionization (APCI) source with direct infusion run in positive ion mode at 5 kV.

General imidazole synthesis[1][2]:

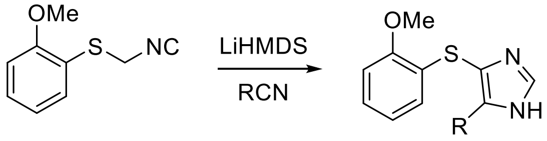

A THF solution of LiHMDS (1.0 M, 1.1 eq.) was added dropwise to a -78 °C, THF solution of Asmic[3] (0.10 M, 1.0 eq.). After 5-10 min, the nitrile or formimidate (1.1 eq.) was added either neat or as a THF solution to the resulting yellow solution. The reaction was maintained at -78 °C for 1-2 h, after which the cooling bath was removed and the reaction was allowed to warm to room temperature. After 1-2 h, saturated, aqueous NH_4Cl (buffered with NH_4OH; pH 7) was added, the phases were separated, and then the aqueous phase was extracted with EtOAc (x3). The combined organic phase was dried (Na_2SO_4), concentrated, and then the crude imidazole was purified on a Reveleris X2 MPLC purification system using a silica gel cartridge.

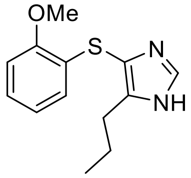

7a

4-((2-Methoxyphenyl)thio)-5-propyl-1H-imidazole (7a) was prepared following the general imidazole procedure using LiHMDS (0.10 mL, 0.10 mmol), Asmic[3] (16 mg, 0.09 mmol), and butyronitrile (7 mg, 0.10 mmol). Purification by MPLC (4 g-silica gel cartridge, EtOAc/hexanes 0:100 to 100:0) furnished 22 mg (99%) of the imidazole **7a** as white solid: mp 134-135 °C; IR (ATR) 2928, 1579 cm^{-1}; ^{1}H NMR (400 MHz, $CDCl_3$) δ 7.56 (s, 1H), 7.06 (td, *J* = 8.2, 7.5, 1.6 Hz, 1H), 6.81 (dd, *J* = 8.2, 1.2 Hz, 1H), 6.76 (td, *J* = 7.6, 1.2 Hz, 1H), 6.61 (dd, *J* = 7.6, 1.6 Hz, 1H), 3.89 (s, 3H), 2.67 – 2.58 (m, 2H), 1.58 (h, *J* = 7.4 Hz, 2H), 0.84 (t, *J* = 7.4 Hz, 3H); ^{13}C NMR (101 MHz, $CDCl_3$) δ 155.2,

136.0, 127.1, 126.2, 126.0, 121.2, 110.3, 55.8, 29.7, 27.2, 22.9, 13.7; HRMS (+APCI) m/z [M+H] calcd for $C_{13}H_{17}N_2OS$ 293.1318; found 293.1322.

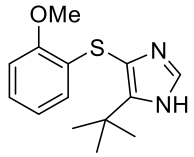
7b

5-(tert-Butyl)-4-((2-methoxyphenyl)thio)-1H-imidazole (7b) was prepared following the general imidazole procedure using LiHMDS (0.26 mL, 0.26 mmol), Asmic[3] (43 mg, 0.24 mmol), and pivalonitrile (22 mg, 0.26 mmol). Purification by MPLC (4-g silica gel cartridge, EtOAc/hexanes 0:100 to 100:0) furnished 48 mg (76%) of the imidazole **7b** as a white solid: mp 178-181 °C; IR (ATR) 2963, 1579, 1476 cm^{-1}; ^{1}H NMR (400 MHz, $CDCl_3$) δ 7.58 (s, 1H), 7.07 (td, *J* = 8.2, 7.6, 1.6 Hz, 1H), 6.82 (dd, *J* = 8.2, 1.2 Hz, 1H), 6.79 (td, *J* = 7.8, 1.2 Hz, 1H), 6.56 (dd, *J* = 7.8, 1.6 Hz, 1H), 3.90 (s, 3H), 1.39 (s, 9H); ^{13}C NMR (101 MHz, $CDCl_3$) δ 155.1, 134.9, 126.4, 126.0, 121.3, 110.3, 55.9, 30.2; HRMS (+APCI) m/z [M+H] calcd for $C_{14}H_{19}N_2OS$ 263.1213; found 263.1215.

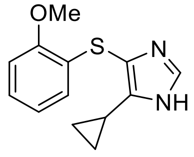
7c

5-Cyclopropyl-4-((2-methoxyphenyl)thio)-1H-imidazole (7c) was prepared following the general imidazole procedure using LiHMDS (0.18 mL, 0.18 mmol), Asmic[3] (30 mg, 0.17 mmol), and cyclopropanecarbonitrile (13 mg, 0.18 mmol). Purification by MPLC (4g-silica gel cartridge, EtOAc/hexanes 0:100 to 100:0) furnished 36 mg (88%) of the imidazole **7c** as a white solid: mp 166 °C; IR (ATR) 3059, 2835, 1579 cm^{-1}; ^{1}H NMR (400 MHz, $CDCl_3$) δ 7.60 (s, 1H), 7.17 – 7.07 (m, 1H), 6.90 – 6.77 (m, 2H), 6.74 – 6.66 (m, 1H), 3.92 (s, 3H), 2.09 – 1.99 (m, 1H), 0.92 – 0.85 (m, 4H); ^{13}C NMR (101 MHz, $CDCl_3$) δ 155.2, 136.0, 126.9, 126.3, 126.0, 121.2, 110.3, 55.8, 22.9, 13.8; HRMS (+APCI) m/z [M+H] calcd for $C_{13}H_{15}N_2OS$ 247.0900; found 247.0901.

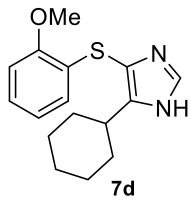

7d

5-Cyclohexyl-4-((2-methoxyphenyl)thio)-1H-imidazole (7d) was prepared following the general imidazole procedure using LiHMDS (0.16 mL, 0.16 mmol), Asmic[3] (26 mg, 0.15 mmol), and cyclohexanecarbonitrile (18 mg, 0.16 mmol). Purification by MPLC (4g-silica gel cartridge, EtOAc/hexanes 38:62 to 100:0) furnished 28 mg (67%) of the imidazole **7d** as a colorless-light yellow oil: IR (ATR) 2852, 1579 cm^{-1}; ^{1}H NMR (400 MHz, $CDCl_3$) δ 7.62 (d, *J* = 0.9 Hz, 1H), 7.18 – 6.99 (m, 1H), 6.84 – 6.80 (m, 1H), 6.77 (tt, *J* = 7.5, 1.1 Hz, 1H), 6.59 (dt, *J* = 7.7, 1.3 Hz, 1H), 2.87 (tt, *J* = 13.0, 12.3, 3.2 Hz, 1H), 1.74 (td, *J* = 13.0, 12.3, 3.2 Hz, 4H), 1.66 (q, *J* = 13.0, 3.2 Hz, 2H), 1.50 (qd, *J* = 13.0, 12.3, 3.2 Hz, 2H), 1.31 (tt, *J* = 13.2, 3.6 Hz, 2H), 1.18 (qt, *J* = 13.2, 12.3, 3.6 Hz, 1H); ^{13}C NMR (101 MHz, $CDCl_3$) δ 155.2, 136.1, 127.0, 126.3, 126.1, 121.2, 110.9, 55.8, 35.1, 32.8, 26.4, 25.8; HRMS (+APCI) m/z [M+H] calcd for $C_{16}H_{20}N_2OS$ 289.1369; found 289.1368.

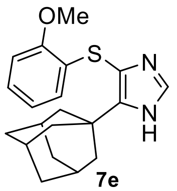

7e

5-(Adamantan-1-yl)-4-((2-methoxyphenyl)thio)-1H-imidazole (7e) was prepared following the general imidazole procedure using LiHMDS (0.14 mL, 0.14 mmol), Asmic[3] (24 mg, 0.13 mmol), and 1-adamantanecarbonitrile (24 mg, 0.15 mmol). Purification by MPLC (4g-silica gel cartridge, EtOAc/hexanes 51:49 to 100:0) furnished 26 mg (57%) of the imidazole **7e** as an off-white solid: mp 204 °C; IR (ATR) 2904, 1666, 1577 cm^{-1}; ^{1}H NMR (400 MHz, $CDCl_3$) δ 7.55 (s, 1H), 7.06 (td, *J* = 8.1, 7.4, 1.6 Hz, 1H), 6.81 (dd, *J* = 8.1, 1.2 Hz, 1H), 6.78 (td, *J* = 7.8, 1.2 Hz, 1H), 6.54 (dd, *J* = 7.8, 1.6 Hz, 1H), 3.89 (s, 3H), 2.08 (d, *J* = 3.0 Hz, 6H), 1.98 (t, *J* = 3.0 Hz, 3H), 1.70 (t, *J* = 3.0 Hz, 6H); ^{13}C NMR (101 MHz, $CDCl_3$) δ 155.1, 135.1, 127.6, 126.3, 125.7, 121.2, 110.2, 55.9, 41.4, 36.5, 34.3, 28.5; HRMS (+APCI) m/z [M+H] calcd for $C_{20}H_{24}N_2OS$ 341.1682; found 341.1681.

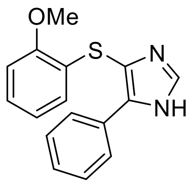

7f

4-((2-Methoxyphenyl)thio)-5-phenyl-1H-imidazole (7f) was prepared following the general imidazole procedure using LiHMDS (0.23 mL, 0.23 mmol), Asmic[3] (39 mg, 0.22 mmol), and benzonitrile (25 mg, 0.24 mmol). Purification by MPLC (4g-silica gel cartridge, EtOAc/hexanes 0:100 to 100:0) furnished 57 mg (93%) of the imidazole **7f** as a white solid: mp 171-174 °C; IR (ATR) 2836, 1578, 1476 cm^{-1}; ^{1}H NMR (400 MHz, $CDCl_3$) δ 7.98 – 7.90 (m, 2H), 7.74 (s, 1H), 7.40 – 7.33 (m, 2H), 7.31 – 7.27 (m, 1H), 7.16 – 7.09 (m, 1H), 6.89 – 6.84 (m, 1H), 6.82 – 6.75 (m, 2H), 3.90 (s, 3H); ^{13}C NMR (101 MHz, $CDCl_3$) δ 136.9, 128.4, 127.7, 127.2, 127.0, 121.5, 110.7, 55.9; HRMS (+APCI) m/z [M+H] calcd for $C_{16}H_{15}N_2OS$ 283.0900; found 283.0901.

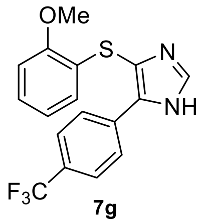

7g

4-((2-Methoxyphenyl)thio)-5-(4-(trifluoromethyl)phenyl)-1H-imidazole (7g) was prepared following the general imidazole procedure using LiHMDS (0.12 mL, 0.12 mmol), Asmic[3] (20 mg, 0.11 mmol), and 4-(trifluoromethyl)benzonitrile (21 mg, 0.12 mmol). Purification by MPLC (4g-silica gel cartridge, EtOAc/hexanes 40:60 to 100:0) furnished 30 mg of the imidazole **7g** as white solid: mp 148-149 °C; IR (ATR) 2937, 1620, 1580 cm^{-1}; ^{1}H NMR (400 MHz, $CDCl_3$) δ 8.13 (d, *J* = 8.2 Hz, 2H), 7.80 (s, 1H), 7.60 (d, *J* = 8.2 Hz, 2H), 7.17 (dddd, *J* = 8.2, 6.9, 2.0, 0.9 Hz, 1H), 6.89 (dt, *J* = 8.2, 1.0 Hz, 1H), 6.81 (tt, *J* = 7.8, 6.9, 1.0 Hz, 1H), 6.78 (ddd, *J* = 7.8, 2.0, 0.7 Hz, 1H), 3.91 (s, 3H); ^{13}C NMR (101 MHz, $CDCl_3$) δ 156.0, 137.3, 129.3 (q, *J* = 32 Hz), 127.6, 127.5, 127.1, 125.6, 125.3 (q, *J* = 3.7 Hz), 124.2, 122.9, 121.6, 110.9, 56.0; HRMS (+APCI) m/z [M+H] calcd for $C_{17}H_{14}F_3N_2OS$ 351.0773; found 351.0777.

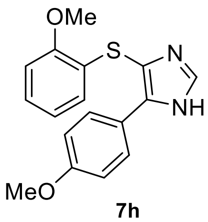

7h

5-(4-Methoxyphenyl)-4-((2-methoxyphenyl)thio)-1H-imidazole (7h) was prepared by the general imidazole procedure using LiHMDS (0.13 mL, 0.13 mmol), Asmic[3] (22 mg, 0.12 mmol), and 4-methoxybenzonitrile (18 mg, 0.14 mmol), with the modification that the reaction was allowed to stir for 3 h at -78 °C before removal from the cooling bath. Purification by MPLC (4g-silica gel cartridge, EtOAc/hexanes 0:100 to 100:0) furnished 25 mg (65%) of the imidazole **7h** as an off-white solid: mp 194-196 °C; IR (ATR) 1577, 1508, 1476 cm^{-1}; ^{1}H NMR (400 MHz, $CDCl_3$) δ 7.87 – 7.82 (m, 2H), 7.70 (s, 1H), 7.11 (ddd, *J* = 8.1, 7.1, 1.9 Hz, 1H), 6.90 – 6.86 (m, 2H), 6.85 (dd, *J* = 8.1, 1.0 Hz, 1H), 6.79 (td, *J* = 7.4, 1.0 Hz, 1H), 6.74 (dd, *J* = 7.4, 1.9 Hz, 1H), 3.90 (s, 3H), 3.79 (s, 3H); ^{13}C NMR (101 MHz, $CDCl_3$) δ 159.2, 155.7, 136.7, 128.3, 126.9, 126.8, 121.5, 113.8, 110.6, 55.9, 55.3; HRMS (+APCI) m/z [M+H] calcd for $C_{17}H_{17}N_2O_2S$ 313.1005; found 313.1012.

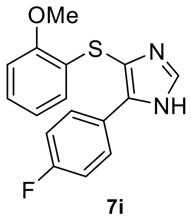

7i

5-(4-Fluorophenyl)-4-((2-methoxyphenyl)thio)-1H-imidazole (7i) was prepared following the general imidazole procedure using LiHMDS (0.26 mL, 0.26 mmol), Asmic[3] (44 mg, 0.25 mmol), and 4-fluorobenzonitrile (33 mg, 0.26 mmol). Purification by MPLC (4g-silica gel cartridge, EtOAc/hexanes 0:100 to 100:0) furnished 50 mg (59%) of the imidazole **7i** as a light orange solid: mp 165 °C; IR (ATR) 1608, 1579, 1506 cm^{-1}; ^{1}H NMR (400 MHz, $CDCl_3$) δ 7.88 – 7.78 (m, 2H), 7.61 (s, 1H), 7.10 (ddd, *J* = 8.2, 7.3, 1.7 Hz, 1H), 7.03 – 6.93 (m, 2H), 6.82 (dd, *J* = 8.2, 1.2 Hz, 1H), 6.77 (td, *J* = 7.6, 1.2 Hz, 1H), 6.70 (dd, *J* = 7.6, 1.7 Hz, 1H), 3.85 (s, 3H); ^{13}C NMR (101 MHz, $CDCl_3$) δ 162.4 (d, $^{1}J_{C-F}$ = 247.1 Hz), 155.8, 137.0, 128.8, 128.8, 127.2, 127.1, 121.5, 115.3 (d, $^{2}J_{C-F}$ = 22 Hz), 110.7, 55.9; HRMS (+APCI) m/z [M+H] calcd for $C_{16}H_{14}FN_2OS$ 301.0805; found 301.0805.

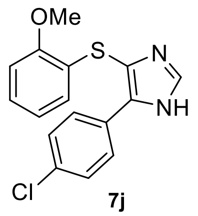

7j

5-(4-Chlorophenyl)-4-((2-methoxyphenyl)thio)-1H-imidazole (7j)[4] was prepared following the general imidazole procedure using LiHMDS (0.16 mL, 0.16 mmol), Asmic[3] (27 mg, 0.15 mmol), and 4-chlorobenzonitrile (23 mg, 0.17 mmol). Purification by MPLC (4g-silica gel cartridge, EtOAc/hexanes 0:100 to 100:0) furnished 38 mg (80%) of the imidazole **7j** as an off-white solid: mp 189-190 °C; IR (ATR) 1579, 1477 cm^{-1}; ^{1}H NMR (400 MHz, $CDCl_3$) δ 7.94 (d, *J* = 8.2 Hz, 2H), 7.78 (s, 1H), 7.35 – 7.29 (m, 2H), 7.16 (td, *J* = 8.1, 7.3, 1.8 Hz, 1H), 6.88 (dd, *J* = 8.1, 1.2 Hz, 1H), 6.80 (td, *J* = 7.5, 1.2 Hz, 1H), 6.75 (dd, *J* = 7.5, 1.8 Hz, 1H), 3.92 (s, 3H); ^{13}C NMR (101 MHz, C_2D_6SO-$CDCl_3$) δ 145.0, 137.9, 132.9, 128.3, 128.1, 126.7, 126.3, 121.6, 110.5, 55.8; HRMS (+APCI) m/z [M+H] calcd for $C_{16}H_{14}ClN_2OS$ 317.0510; found 317.0514.

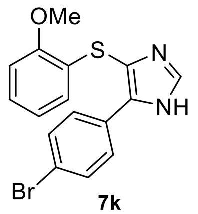

7k

5-(4-Bromophenyl)-4-((2-methoxyphenyl)thio)-1H-imidazole (7k)[4] was prepared following the general imidazole procedure using LiHMDS (0.19 mL, 0.19 mmol), Asmic[3] (32 mg, 0.18 mmol), and 4-bromobenzonitrile (36 mg, 0.19 mmol). Purification by MPLC (4g-silica gel cartridge, EtOAc/hexanes 0:100 to 100:0) afforded 39 mg (61%) of the imidazole **7k** as an off-white solid: mp 193 °C; IR (ATR) 1580, 1475 cm^{-1}; ^{1}H NMR (400 MHz, $CDCl_3$) δ 7.92 – 7.86 (m, 2H), 7.80 (s, 1H), 7.52 – 7.43 (m, 2H), 7.16 (ddd, *J* = 8.3, 7.2, 1.8 Hz, 1H), 6.88 (dd, *J* = 8.3, 1.2 Hz, 1H), 6.80 (td, *J* = 7.7, 7.2, 1.2 Hz, 1H), 6.75 (dd, *J* = 7.7, 1.8 Hz, 1H), 3.92 (s, 3H); ^{13}C NMR (101 MHz, $CDCl_3$) δ 155.9, 137.0, 131.5, 128.5, 127.5, 127.4, 121.7, 121.6, 110.8, 56.0; HRMS (+APCI) m/z [M+H] calcd for $C_{16}H_{14}BrN_2OS$ 361.0005; found 361.0011.

3-(4-((2-Methoxyphenyl)thio)-1H-imidazol-5-yl)-1-methyl-1H-indole

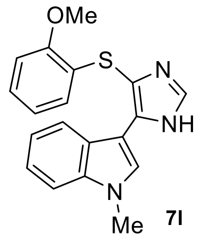
7l

(7l) was prepared following the general imidazole procedure using LiHMDS (0.23 mL, 0.23 mmol), Asmic[3] (39 mg, 0.22 mmol), and 1-methylindole-3-carbonitrile (38 mg, 0.24 mmol). Purification by MPLC (4g-silica gel cartridge, EtOAc/hexanes 43:57 to 100:0) furnished 58 mg (79%) of the imidazole **7l** as a yellow-orange solid: mp 214 °C; IR (ATR) 2161, 2031, 1577, 1474 cm^{-1}; ^{1}H NMR (400 MHz, $CDCl_3$) δ 8.13 (br s, 1H), 7.84 (s, 1H), 7.55 (s, 1H), 7.36 – 7.29 (m, 1H), 7.29 – 7.24 (m, 3H), 7.23 – 7.16 (m, 1H), 7.14 – 7.04 (m, 1H), 6.88 – 6.84 (m, 1H), 6.81 – 6.74 (m, 1H), 3.93 (s, 3H), 3.76 (s, 3H); ^{13}C NMR (101 MHz, $CDCl_3$) δ 126.7, 121.5, 110.5, 55.9, 33.0; HRMS (+APCI) m/z [M+H] calcd for $C_{19}H_{18}N_3OS$ 336.1165; found 336.1167.

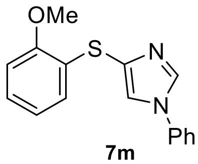
7m

4-((2-Methoxyphenyl)thio)-1-phenyl-1H-imidazole (7m) was prepared following general imidazole procedure using LiHMDS (0.81 mL, 0.81 mmol), Asmic[3] (132 mg, 0.74 mmol), and ethyl-N-phenylformimidate (121 mg, 0.81 mmol). Purification by MPLC (4g-silica gel cartridge, EtOAc/hexanes 0:100 to 40:60) afforded 142 mg (69%) of the imidazole **7m** as an orange solid: mp 88 °C; IR (ATR)2 1577 cm^{-1}; ^{1}H NMR (400 MHz, $CDCl_3$) δ 7.97 (d, *J* = 1.4 Hz, 1H), 7.52 (d, *J* = 1.4 Hz, 1H), 7.51 – 7.46 (m, 2H), 7.41 – 7.36 (m, 3H), 7.14 – 7.09 (m, 1H), 7.08 – 7.04 (m, 1H), 6.87 – 6.80 (m, 2H), 3.91 (s, 3H); ^{13}C NMR (101 MHz, $CDCl_3$) δ 155.6, 136.9, 136.8, 131.5, 130.0, 127.9, 127.8, 126.6, 126.3, 123.8, 121.3, 121.2, 110.5, 55.9; HRMS (+APCI) m/z [M+H] calcd for $C_{16}H_{15}N_2OS$ 283.0900; found 283.0900.

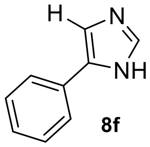
8f

5-Phenyl-1H-imidazole (8f). An aqueous slurry of Raney Nickel (50% (w/w) 2.5 mL) was added to a methanolic solution (20 mL) of 4-((2-methoxyphenyl)thio)-5-phenyl-1H-imidazole (**7f**, 82 mg, 0.29 mmol). After 35 min, the slurry was filtered through celite and then washed with several portions of methanol until the imidazole was not able to be detected in the filtrate by TLC. The combined filtrate was filtered through a pad of silica, the solvent was evaporated *in-vacuo*, and then the aqueous phase was extracted with EtOAc (x3). The combined organic layers were

dried with (Na_2SO_4), concentrated, and purified by MPLC (4-g silica gel cartridge, EtOAc/hexanes 10:90 to 100:0) to furnish 27.0 mg (64%) of the imidazole **8f** as a colorless solid with spectral properties consistent with those exhibited by material previously synthesized.[5]

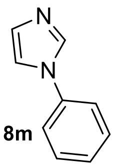

8m

1-Phenyl-1H-imidazole (8m). A methanolic solution (25 mL) of 4-((2-methoxyphenyl)thio)-1-phenyl-1H-imidazole (**7m**, 98 mg, 0.35 mmol) was added to an aqueous slurry of Raney Nickel (50% (w/w) 2.5 mL). After 15 min the slurry was filtered through celite washing with several portions of methanol until the imidazole was not able to be detected by TLC in the filtrate. The methanol was evaporated *in-vacuo*, and then largely aqueous phase was extracted with EtOAc (x3). The combined organic phase was dried (Na_2SO_4), concentrated, and purified by MPLC (4-g silica gel cartridge, EtOAc/hexanes 10:90 to 100:0) to furnish 29.0 mg (58%) of the imidazole **8m** as a colorless amorphous solid with spectral properties identical to that exhibited by material previously synthesized:[6] ^{1}H NMR (400 MHz, $CDCl_3$) δ 7.86 (s, 1H), 7.53 – 7.44 (m, 2H), 7.43 – 7.33 (m, 3H), 7.29 (s, 1H), 7.21 (s, 1H); ^{13}C NMR (126 MHz, $CDCl_3$) δ 137.4, 135.6, 130.4, 129.9, 127.5, 121.5, 118.2.

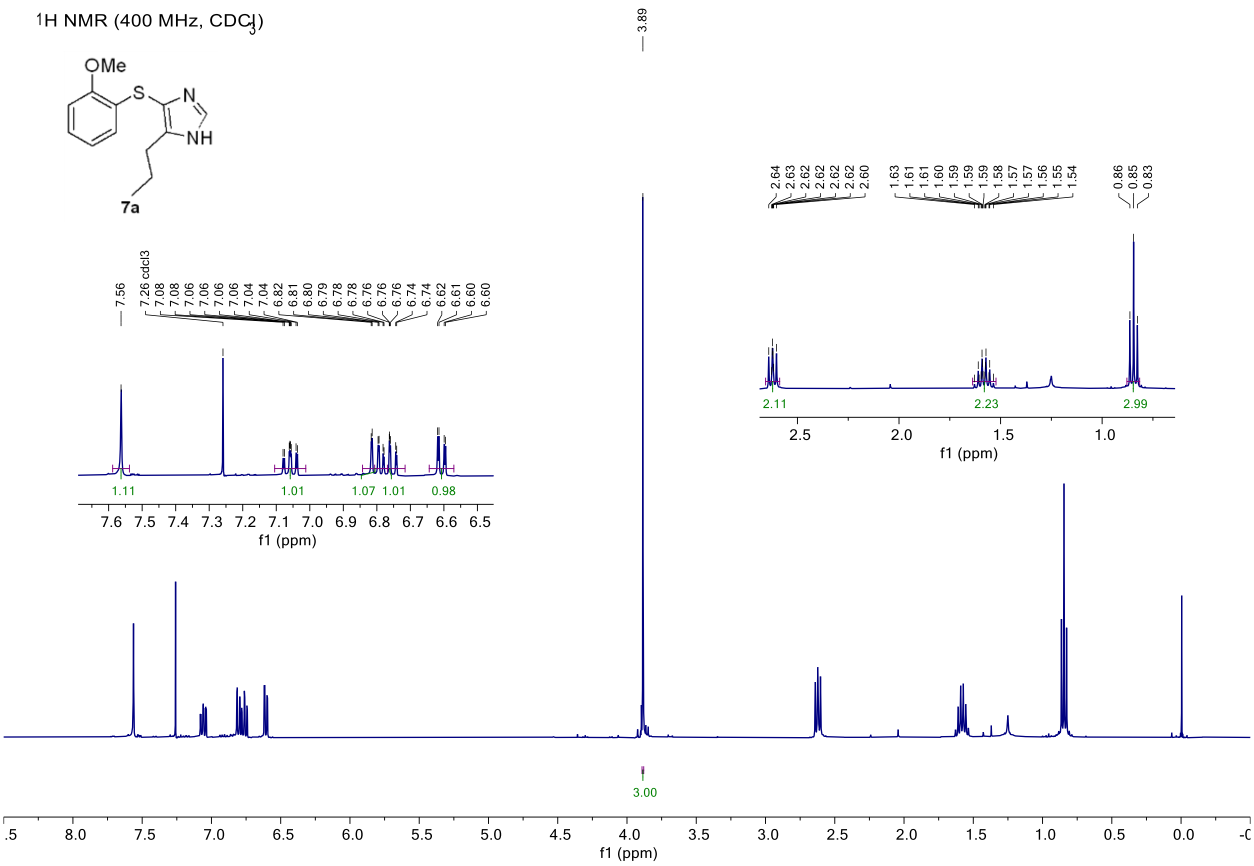
1H NMR (400 MHz, CDCl3)
OMe
S
N
NH
7a
f1 (ppm)

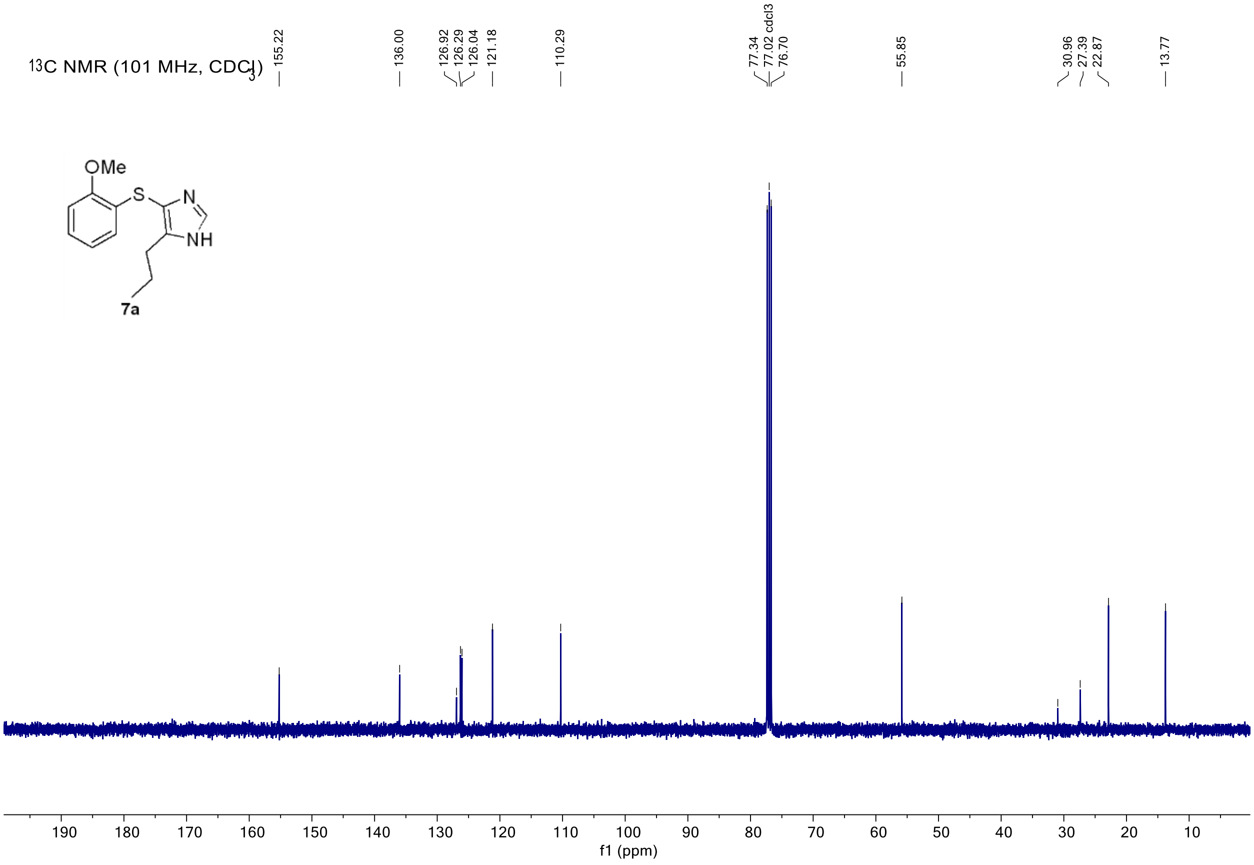
13C NMR (101 MHz, CDCl3)
OMe
S
N
NH
7a
f1 (ppm)

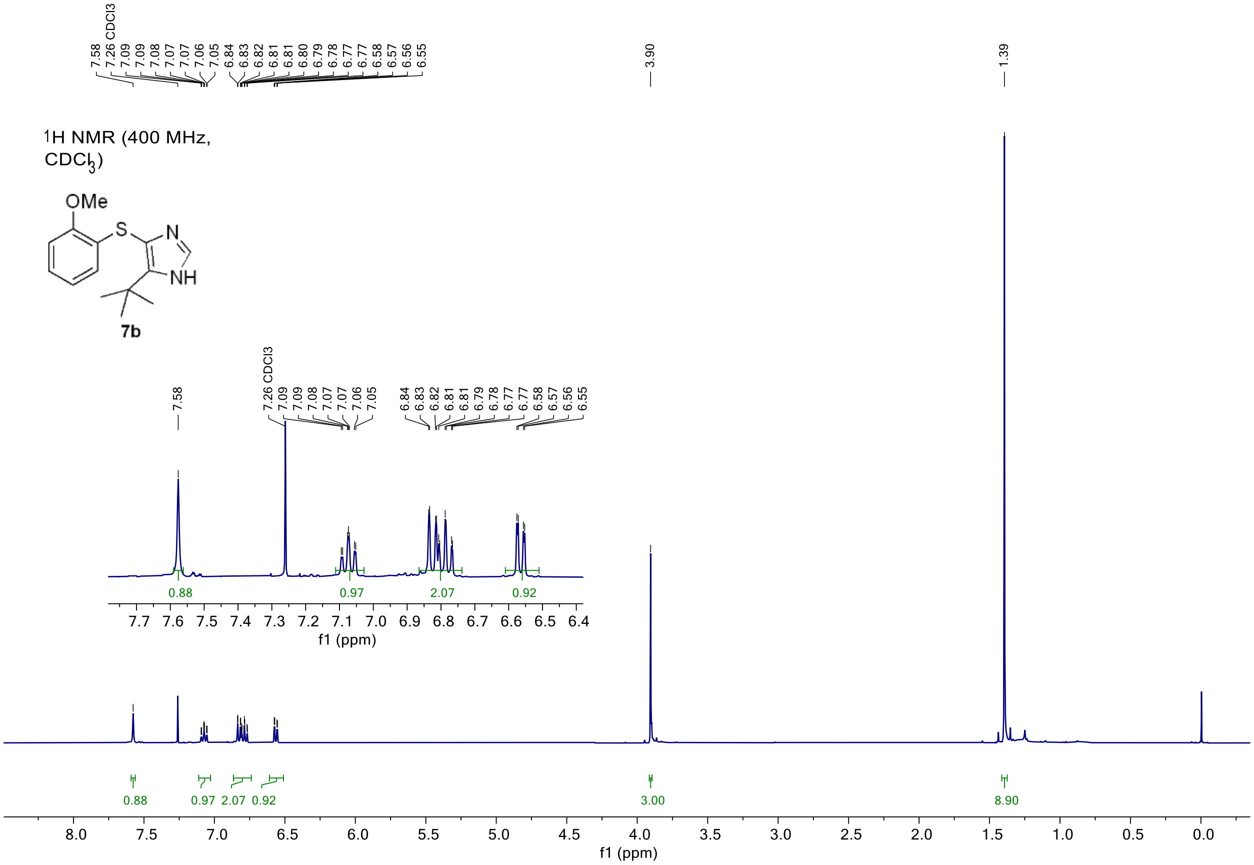
1H NMR (400 MHz, CDCl3)
7.58
7.26 CDCl3
7.09
7.09
7.08
7.07
7.07
7.06
7.05
6.84
6.83
6.82
6.81
6.81
6.80
6.79
6.78
6.77
6.77
6.58
6.57
6.56
6.55
3.90
1.39
OMe
S
N
NH
7b
0.88
0.97
2.07
0.92
3.00
8.90
f1 (ppm)

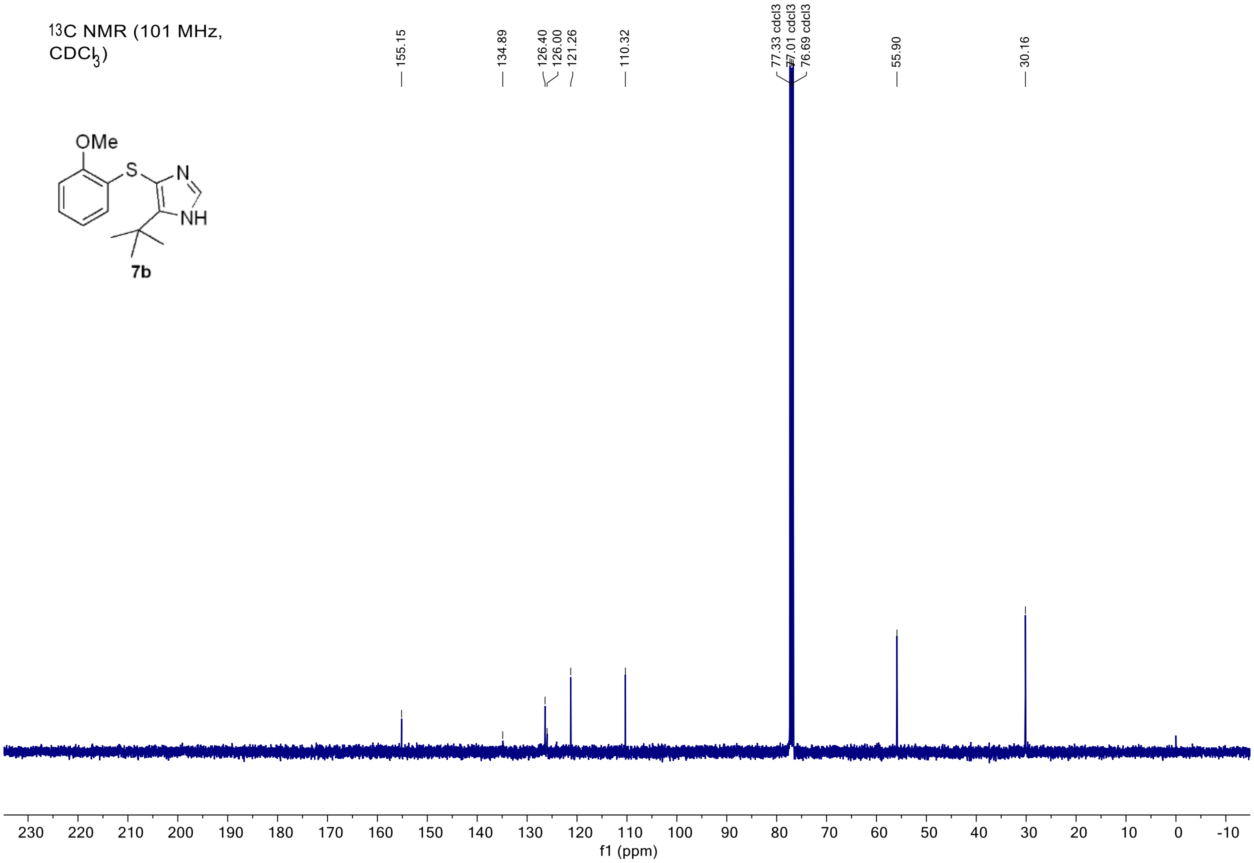
13C NMR (101 MHz, CDCl3)
155.15
134.89
126.40
126.00
121.26
110.32
77.33 cdcl3
77.01 cdcl3
76.69 cdcl3
55.90
30.16
OMe
S
N
NH
7b
f1 (ppm)

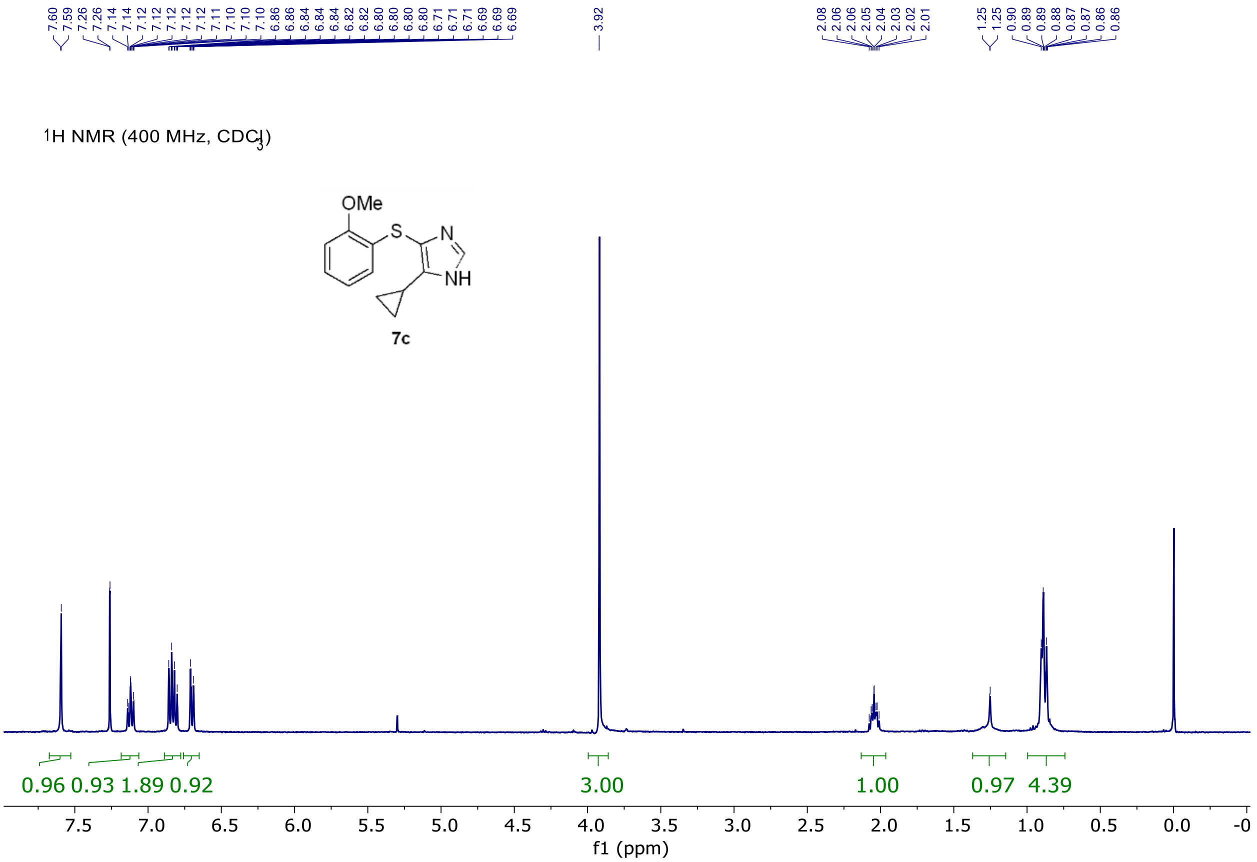

1H NMR (400 MHz, CDCl3)
OMe
S
N
NH
7c
7.60 7.59 7.26 7.14 7.12 7.12 7.12 7.12 7.11 7.10 7.10 6.86 6.86 6.84 6.84 6.84 6.82 6.82 6.80 6.80 6.80 6.80 6.71 6.71 6.69 6.69
3.92
2.08 2.06 2.06 2.05 2.04 2.03 2.02 2.01
1.25 1.25 0.90 0.89 0.89 0.88 0.87 0.87 0.86 0.86
0.96 0.93 1.89 0.92
3.00
1.00
0.97 4.39
f1 (ppm)

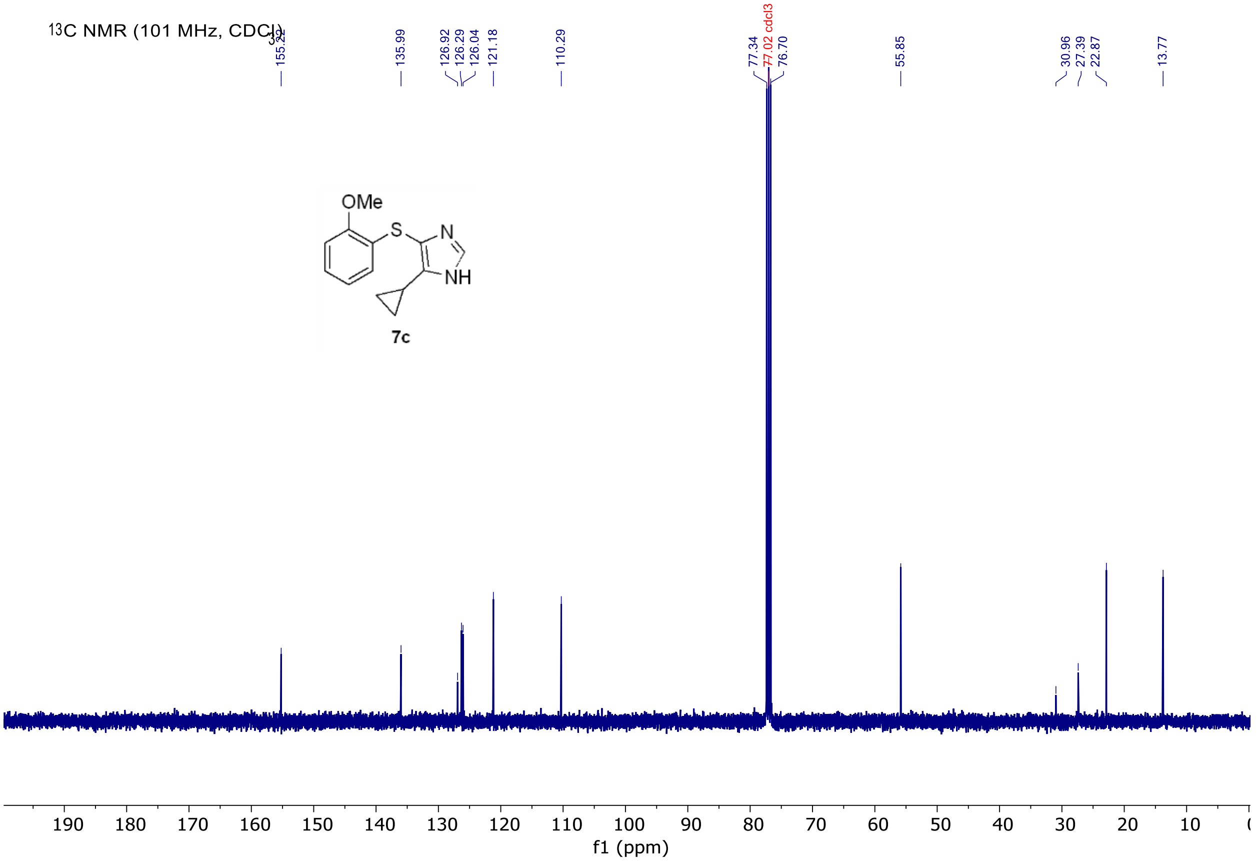

13C NMR (101 MHz, CDCl3)
135.99
126.92 126.29 126.04 121.18
110.29
77.34 77.02 cdcl3 76.70
55.85
30.96 27.39 22.87
13.77
OMe
S
N
NH
7c
f1 (ppm)

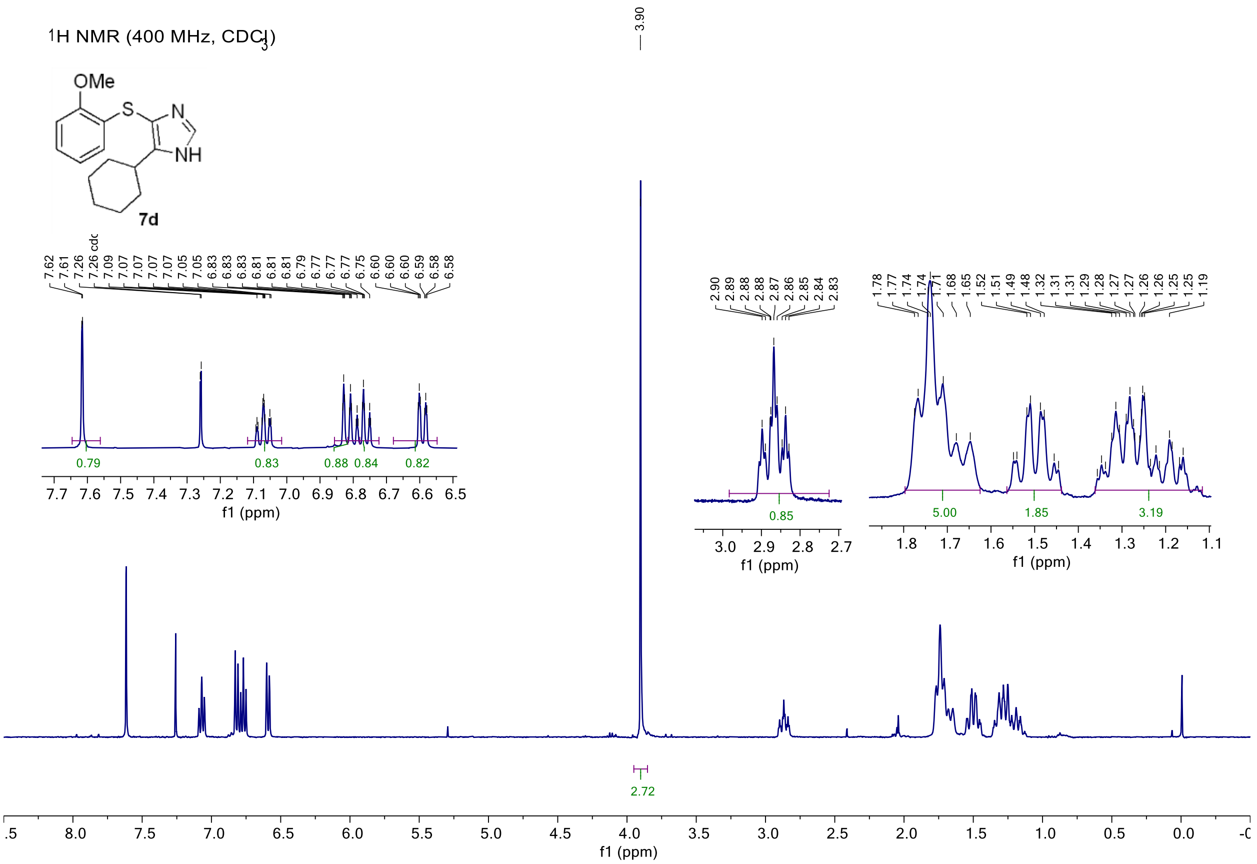
1H NMR (400 MHz, CDCl3)
OMe
S
N
NH
7d
3.90
0.79
0.83
0.88
0.84
0.82
0.85
5.00
1.85
3.19
2.72
f1 (ppm)

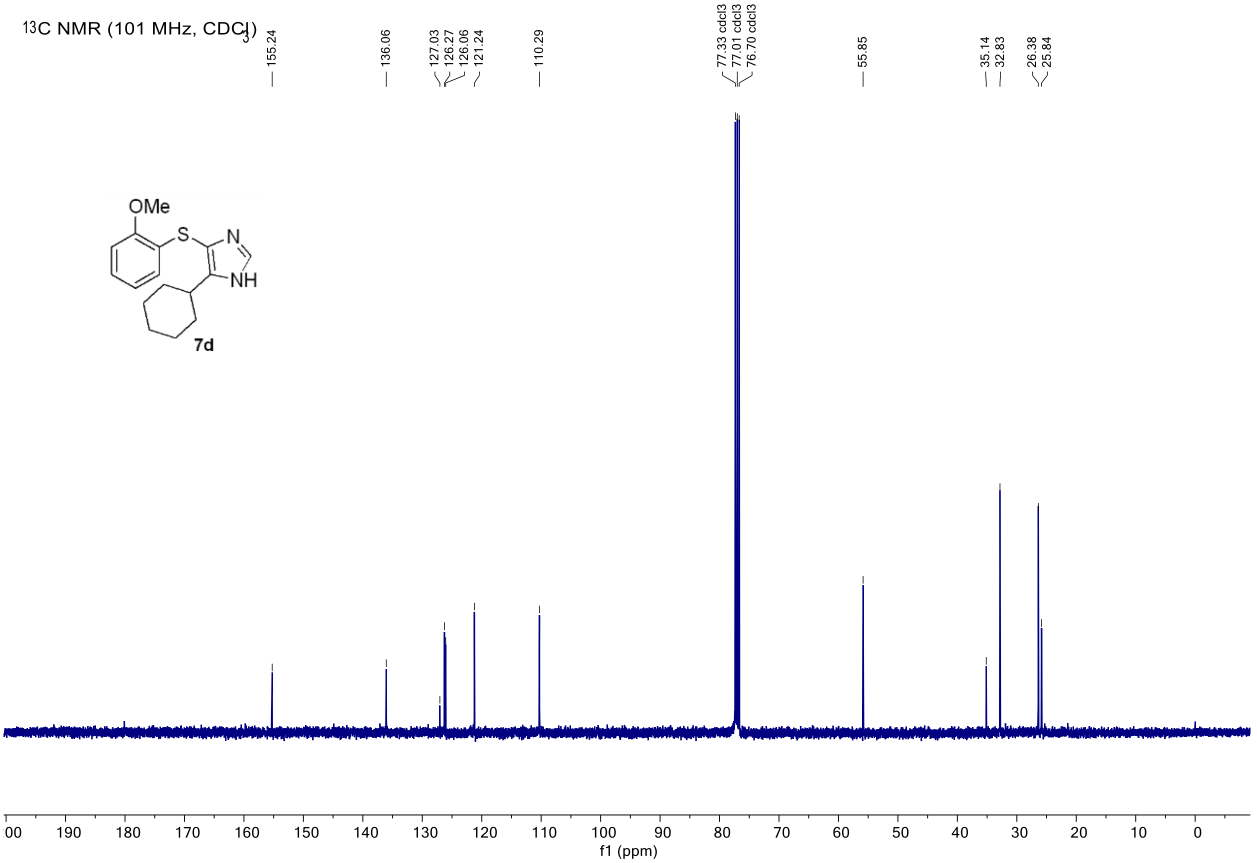
13C NMR (101 MHz, CDCl3)
155.24
136.06
127.03
126.27
126.06
121.24
110.29
77.33 cdcl3
77.01 cdcl3
76.70 cdcl3
55.85
35.14
32.83
26.38
25.84
OMe
S
N
NH
7d
f1 (ppm)

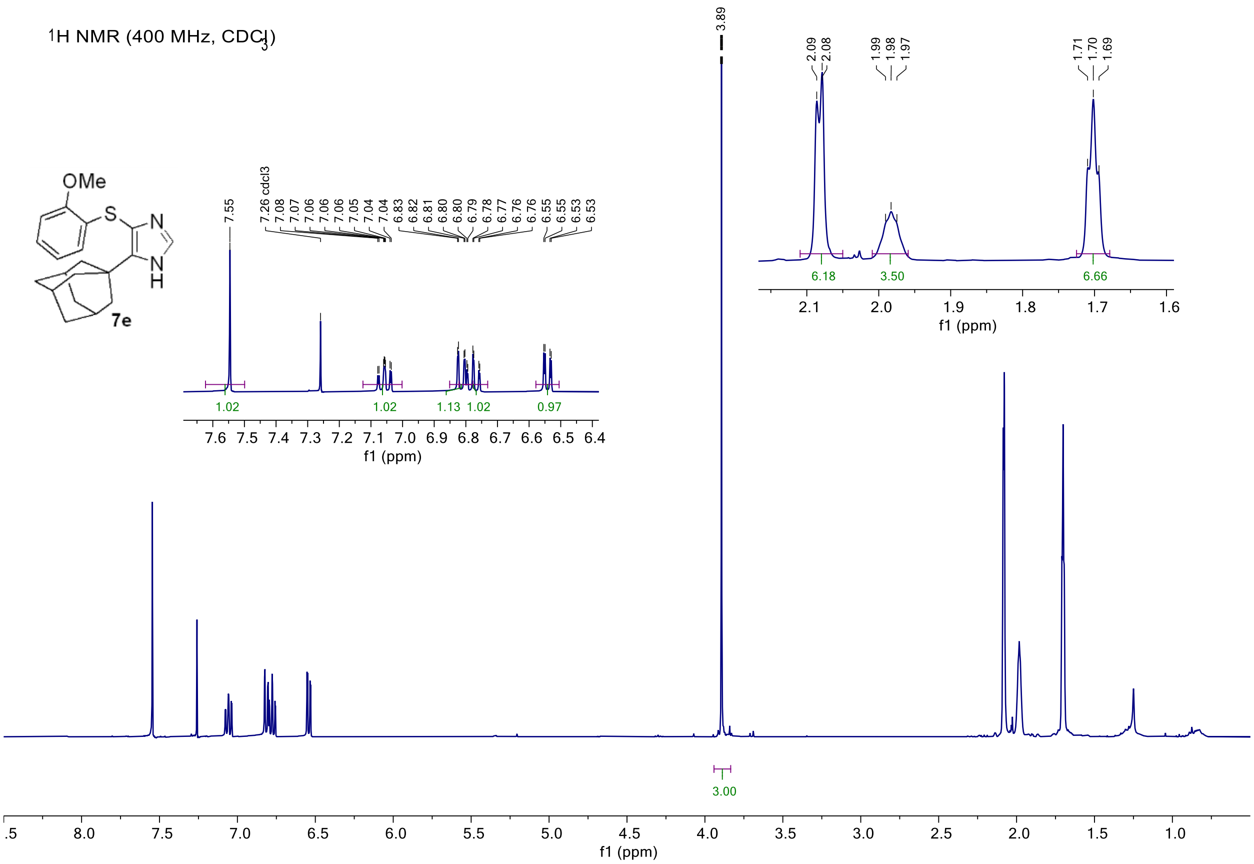
1H NMR (400 MHz, CDCl3)
OMe
S
N
N
H
7e
3.89
2.09 2.08
1.99 1.98 1.97
1.71 1.70 1.69
6.18
3.50
6.66
7.55
7.26 cdcl3
1.02
1.02
1.13 1.02
0.97
3.00
f1 (ppm)

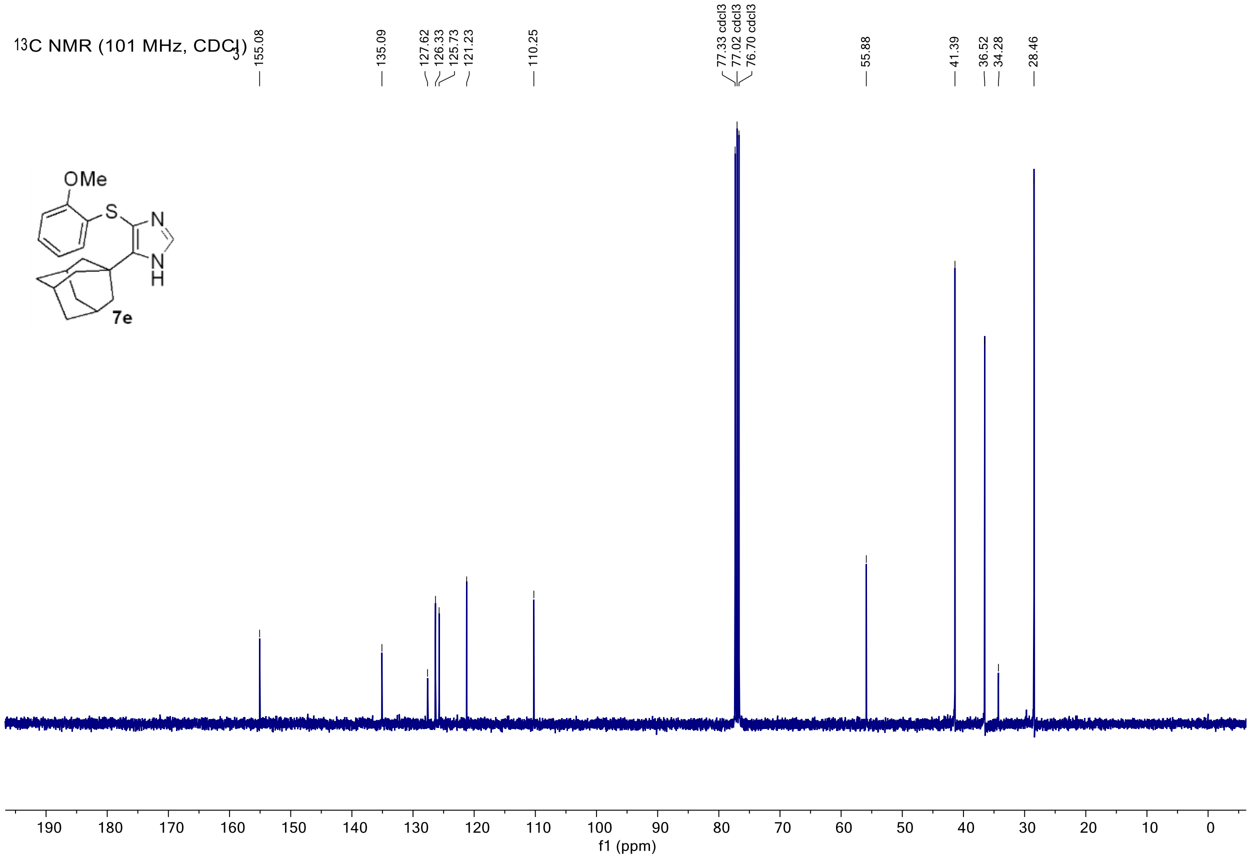
13C NMR (101 MHz, CDCl3)
155.08
135.09
127.62
126.33
125.73
121.23
110.25
77.33 cdcl3
77.02 cdcl3
76.70 cdcl3
55.88
41.39
36.52
34.28
28.46
OMe
S
N
N
H
7e
f1 (ppm)

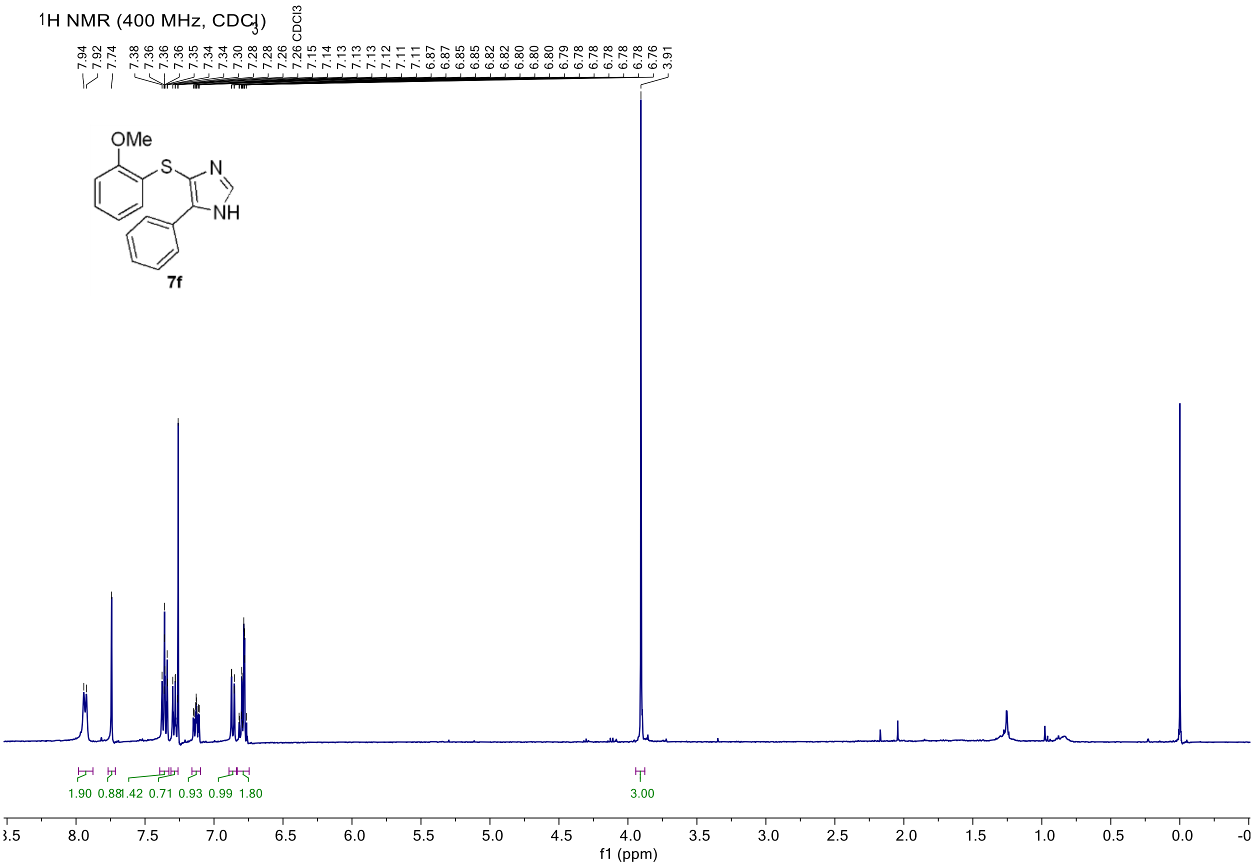
1H NMR (400 MHz, CDCl3)
7.94
7.92
7.74
7.38
7.36
7.36
7.36
7.35
7.34
7.34
7.30
7.28
7.28
7.26
7.26 CDCl3
7.15
7.14
7.13
7.13
7.13
7.12
7.11
7.11
6.87
6.87
6.85
6.85
6.82
6.82
6.80
6.80
6.79
6.78
6.78
6.78
6.78
6.78
6.76
3.91
OMe
S
N
NH
7f
1.90
0.88
1.42
0.71
0.93
0.99
1.80
3.00
f1 (ppm)

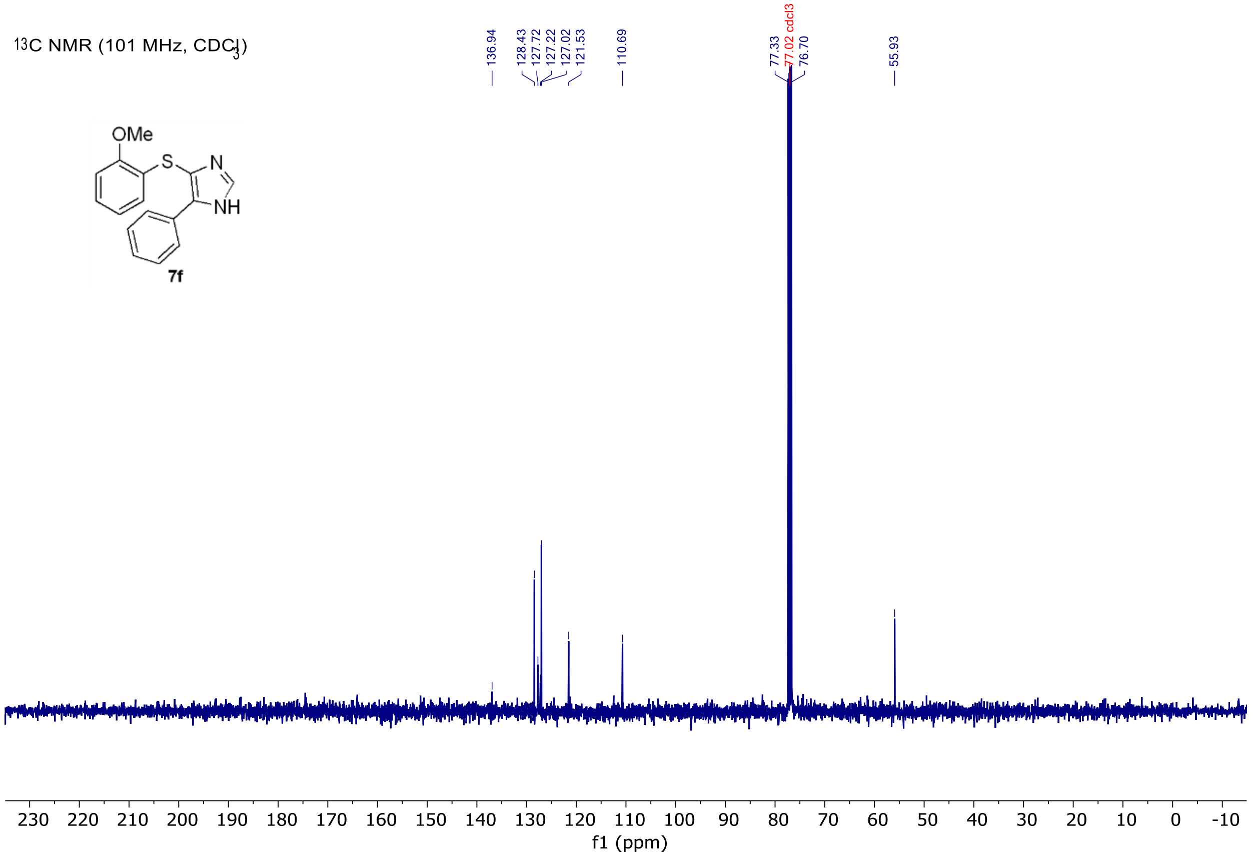
13C NMR (101 MHz, CDCl3)
136.94
128.43
127.72
127.22
127.02
121.53
110.69
77.33
77.02 cdcl3
76.70
55.93
OMe
S
N
NH
7f
f1 (ppm)

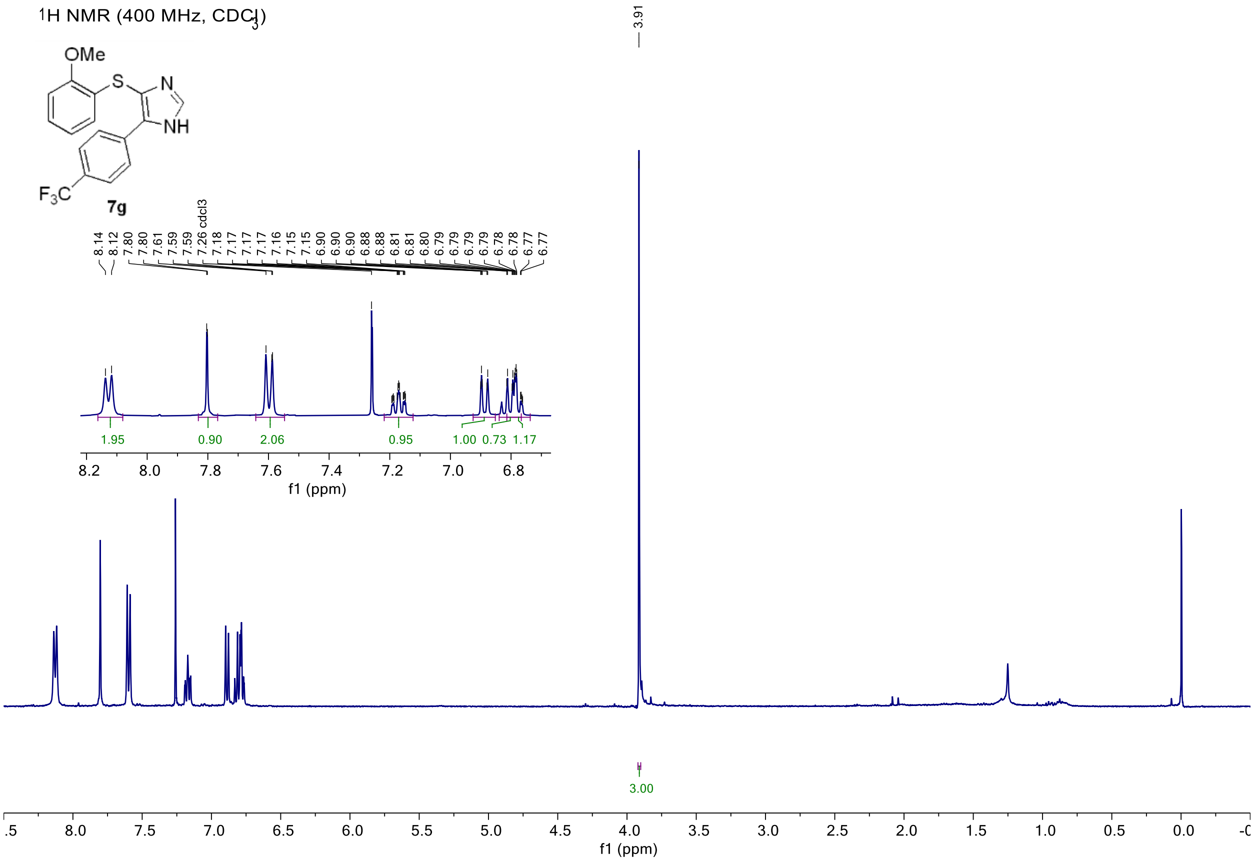
1H NMR (400 MHz, CDCl3)
OMe
S
N
NH
F3C
7g
3.91
1.95
0.90
2.06
0.95
1.00
0.73
1.17
3.00
f1 (ppm)

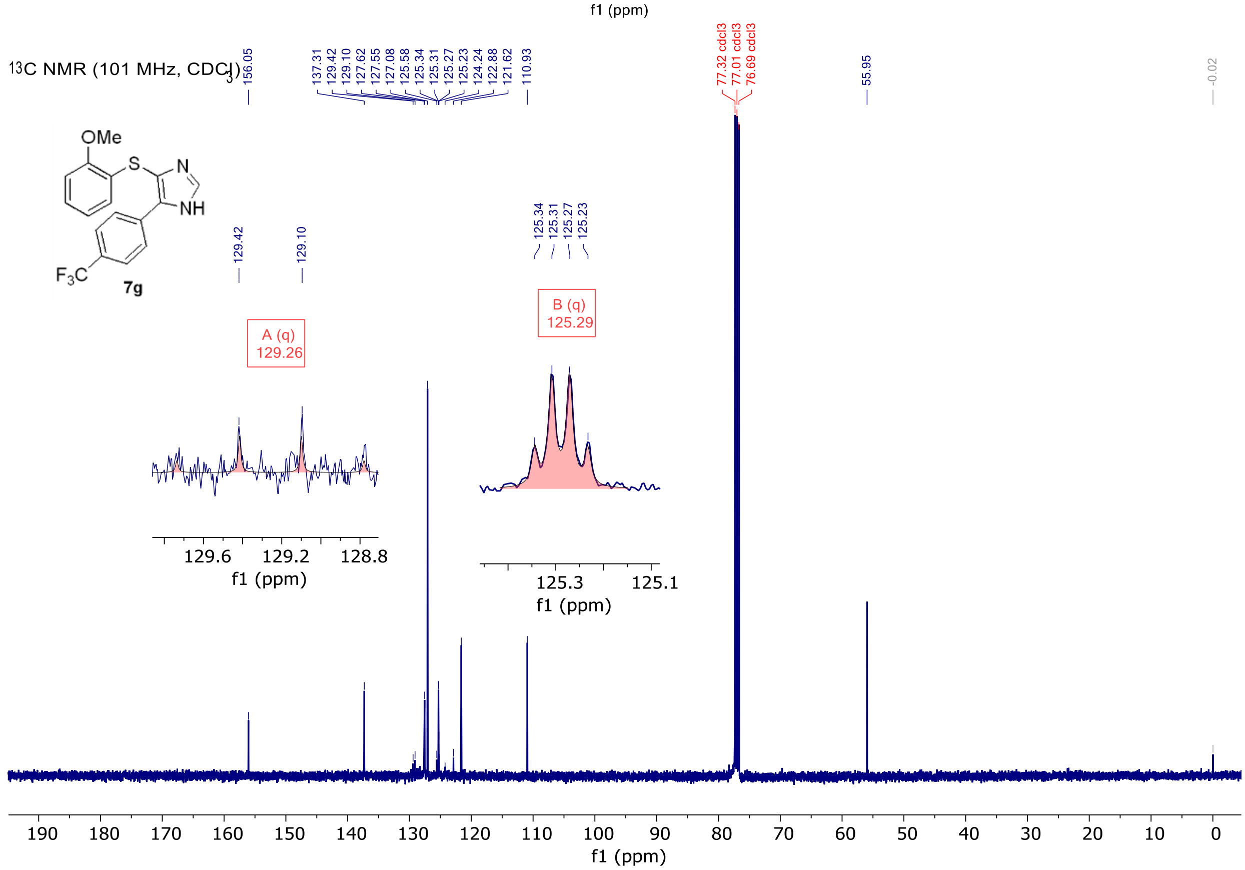
13C NMR (101 MHz, CDCl3)
OMe
S
N
NH
F3C
7g
156.05
137.31
129.42
129.10
127.62
127.55
127.08
125.58
125.34
125.31
125.27
125.23
124.24
122.88
121.62
110.93
77.32 cdcl3
77.01 cdcl3
76.69 cdcl3
55.95
-0.02
A (q)
129.26
B (q)
125.29
f1 (ppm)

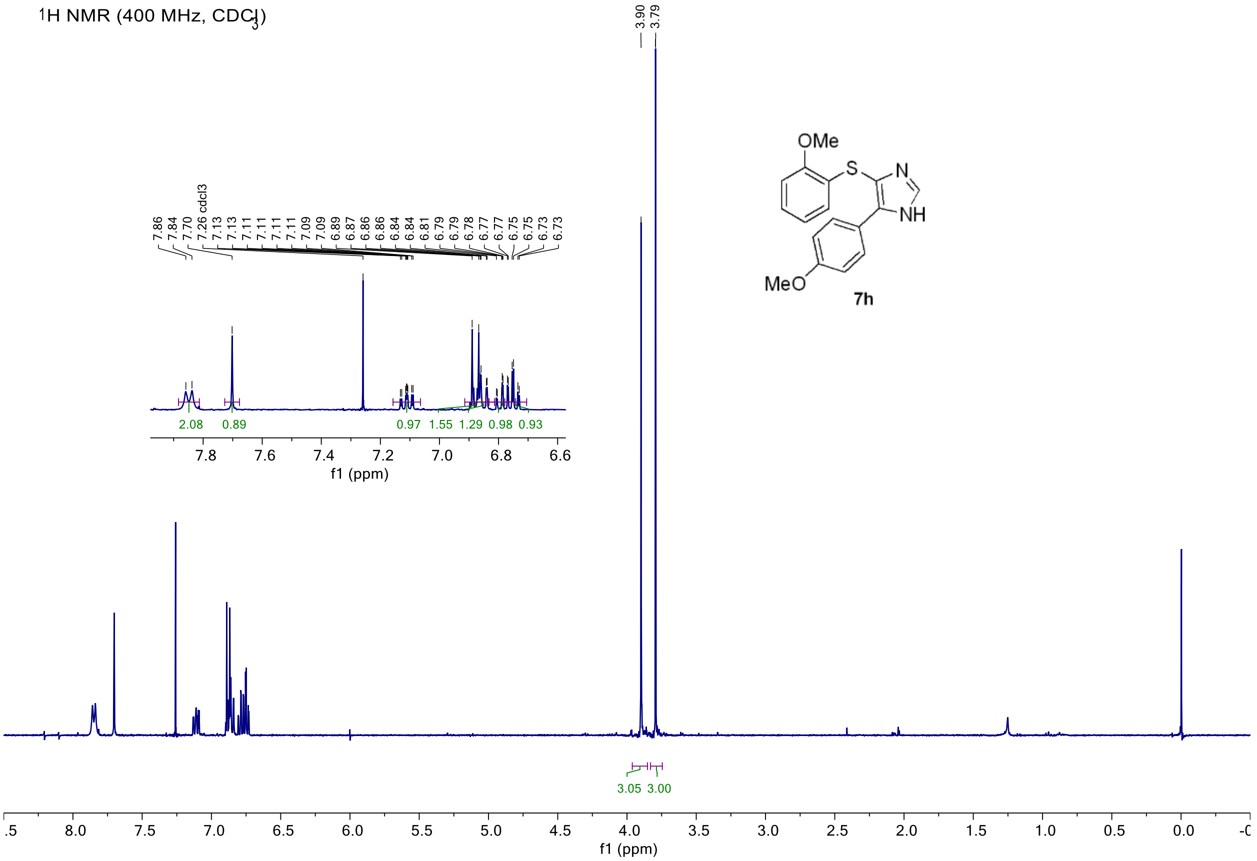
1H NMR (400 MHz, CDCl3)
3.90
3.79
7.86 7.84 7.70 7.26 cdcl3 7.13 7.13 7.11 7.11 7.11 7.11 7.09 7.09 6.89 6.87 6.86 6.86 6.84 6.84 6.81 6.79 6.79 6.78 6.77 6.77 6.75 6.75 6.73 6.73
2.08 0.89 0.97 1.55 1.29 0.98 0.93
f1 (ppm)
3.05 3.00
OMe
S
N
NH
MeO
7h
f1 (ppm)

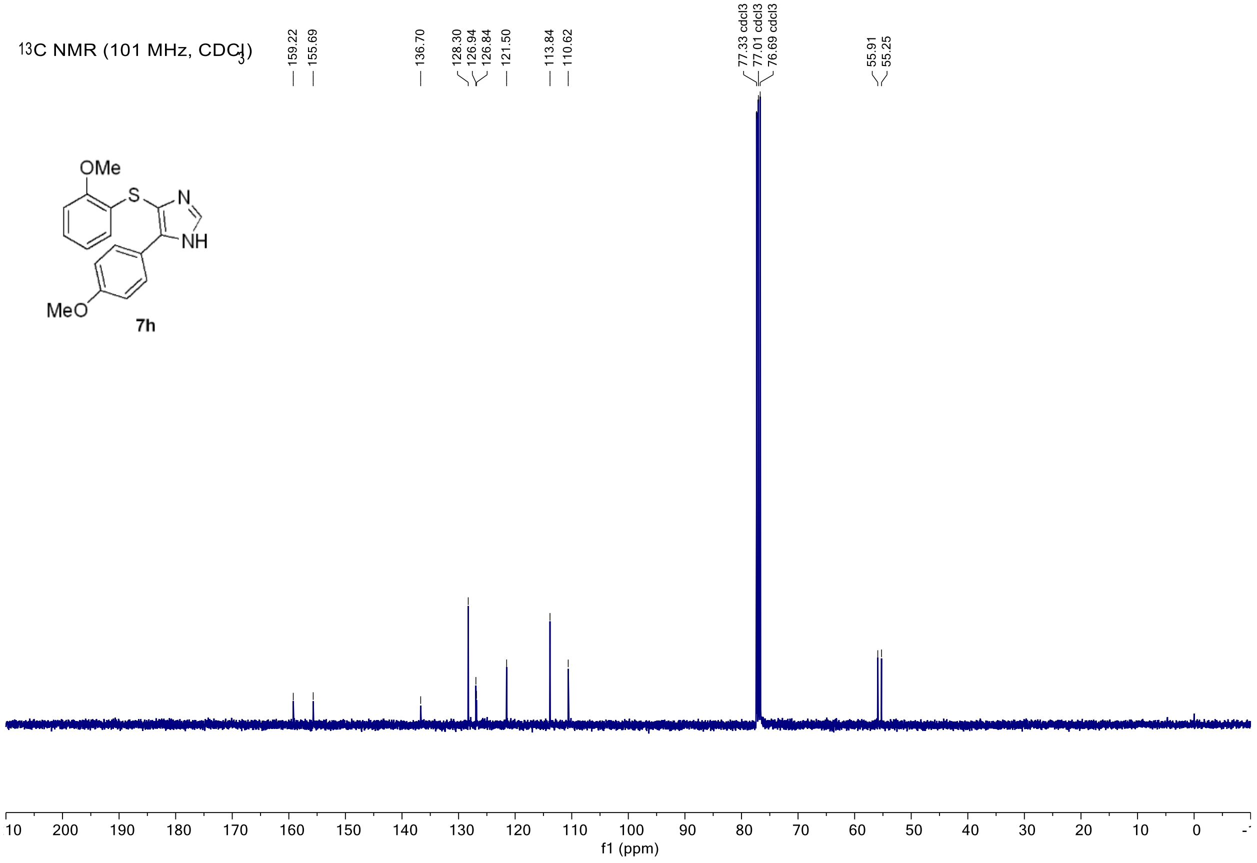
13C NMR (101 MHz, CDCl3)
159.22
155.69
136.70
128.30
126.94
126.84
121.50
113.84
110.62
77.33 cdcl3
77.01 cdcl3
76.69 cdcl3
55.91
55.25
OMe
S
N
NH
MeO
7h
f1 (ppm)

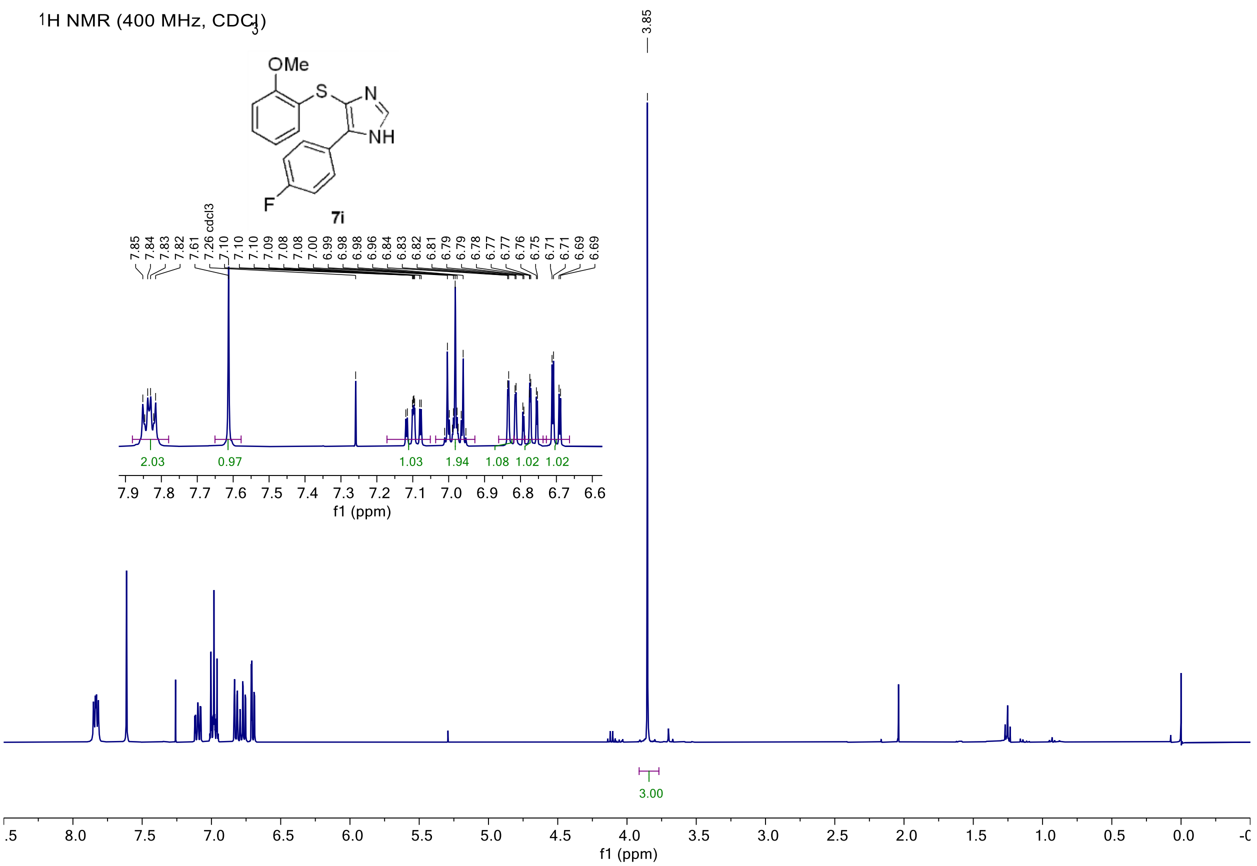
1H NMR (400 MHz, CDCl3)
OMe
S
N
NH
F
7i
7.85 7.84 7.83 7.82 7.61 7.26 cdcl3 7.10 7.10 7.10 7.09 7.08 7.08 7.00 6.99 6.98 6.98 6.96 6.84 6.83 6.82 6.81 6.79 6.79 6.78 6.77 6.77 6.76 6.75 6.71 6.71 6.69 6.69
2.03 0.97 1.03 1.94 1.08 1.02 1.02
f1 (ppm)
3.85
3.00
f1 (ppm)

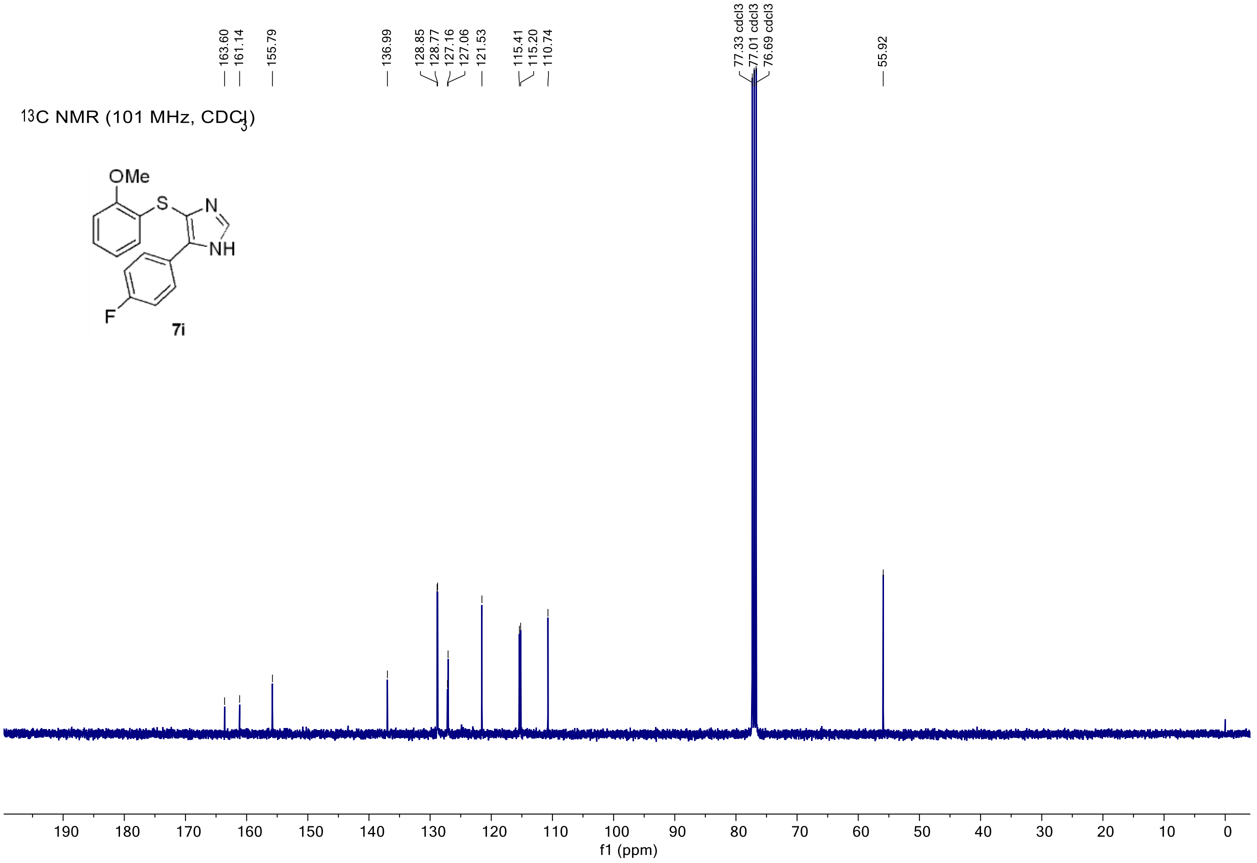
163.60 161.14 155.79 136.99 128.85 128.77 127.16 127.06 121.53 115.41 115.20 110.74 77.33 cdcl3 77.01 cdcl3 76.69 cdcl3 55.92
13C NMR (101 MHz, CDCl3)
OMe
S
N
NH
F
7i
f1 (ppm)

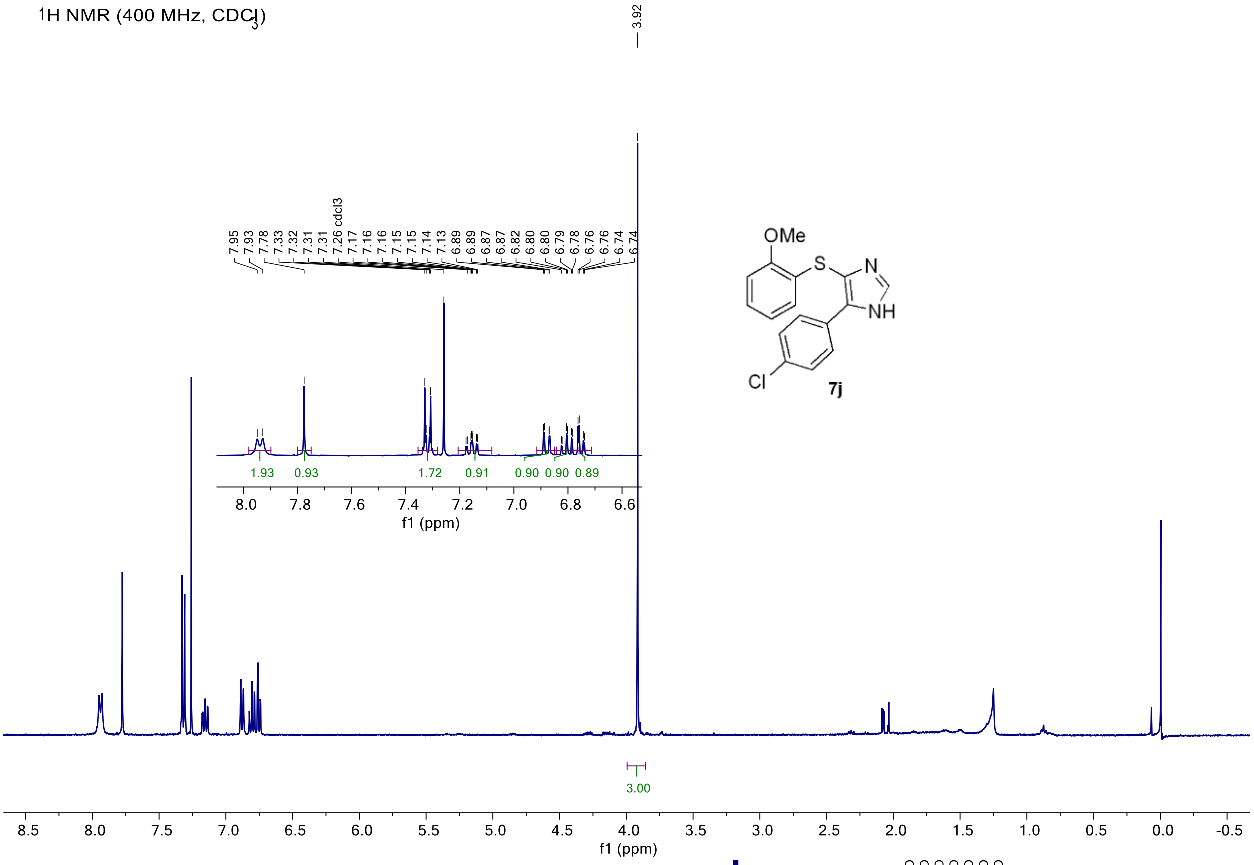
1H NMR (400 MHz, CDCl3)
3.92
7.95 7.93 7.78 7.33 7.32 7.31 7.31 7.26 cdcl3 7.17 7.16 7.16 7.15 7.15 7.14 7.13 6.89 6.89 6.87 6.87 6.82 6.80 6.80 6.79 6.78 6.76 6.76 6.74 6.74
1.93 0.93 1.72 0.91 0.90 0.90 0.89
3.00
OMe
S
N
NH
Cl
7j
f1 (ppm)

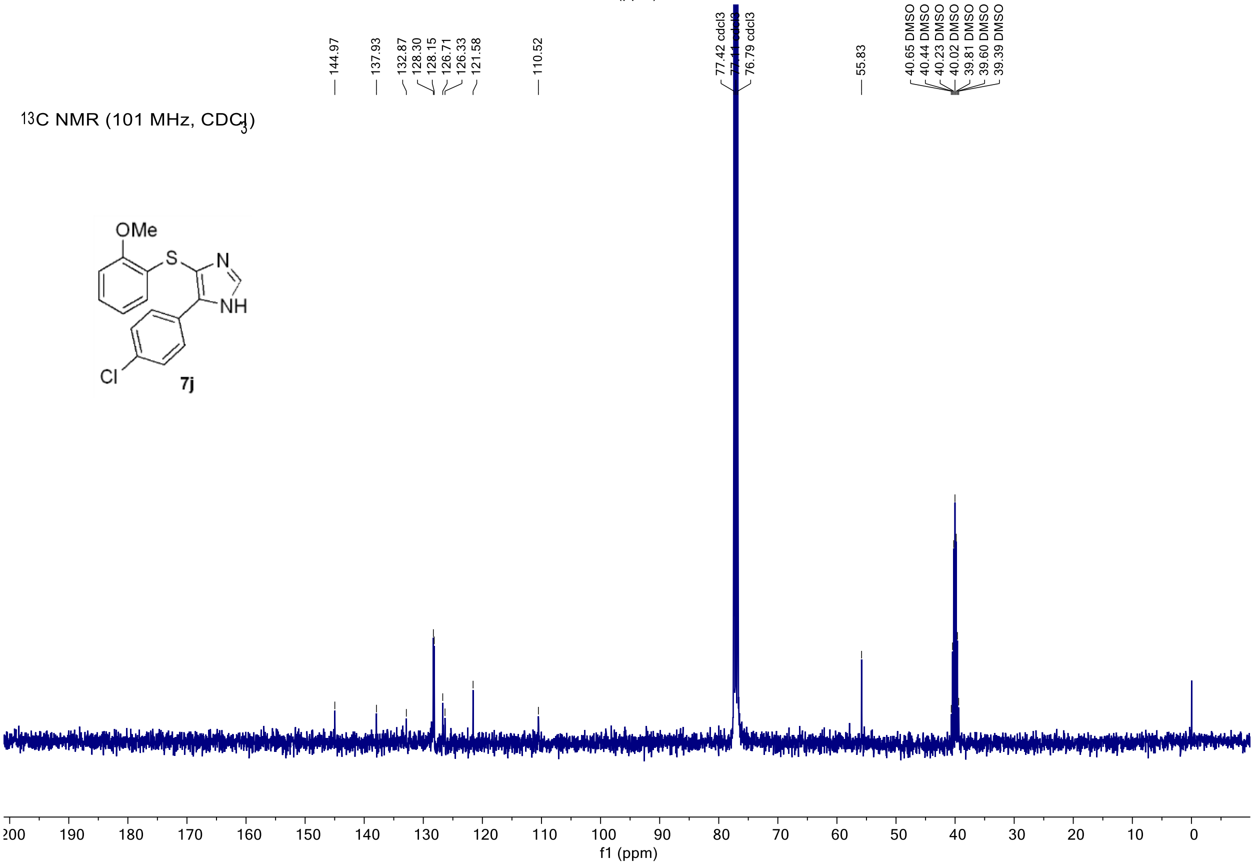
13C NMR (101 MHz, CDCl3)
144.97
137.93
132.87
128.30
128.15
126.71
126.33
121.58
110.52
77.42 cdcl3
76.79 cdcl3
55.83
40.65 DMSO
40.44 DMSO
40.23 DMSO
40.02 DMSO
39.81 DMSO
39.60 DMSO
39.39 DMSO
OMe
S
N
NH
Cl
7j
f1 (ppm)

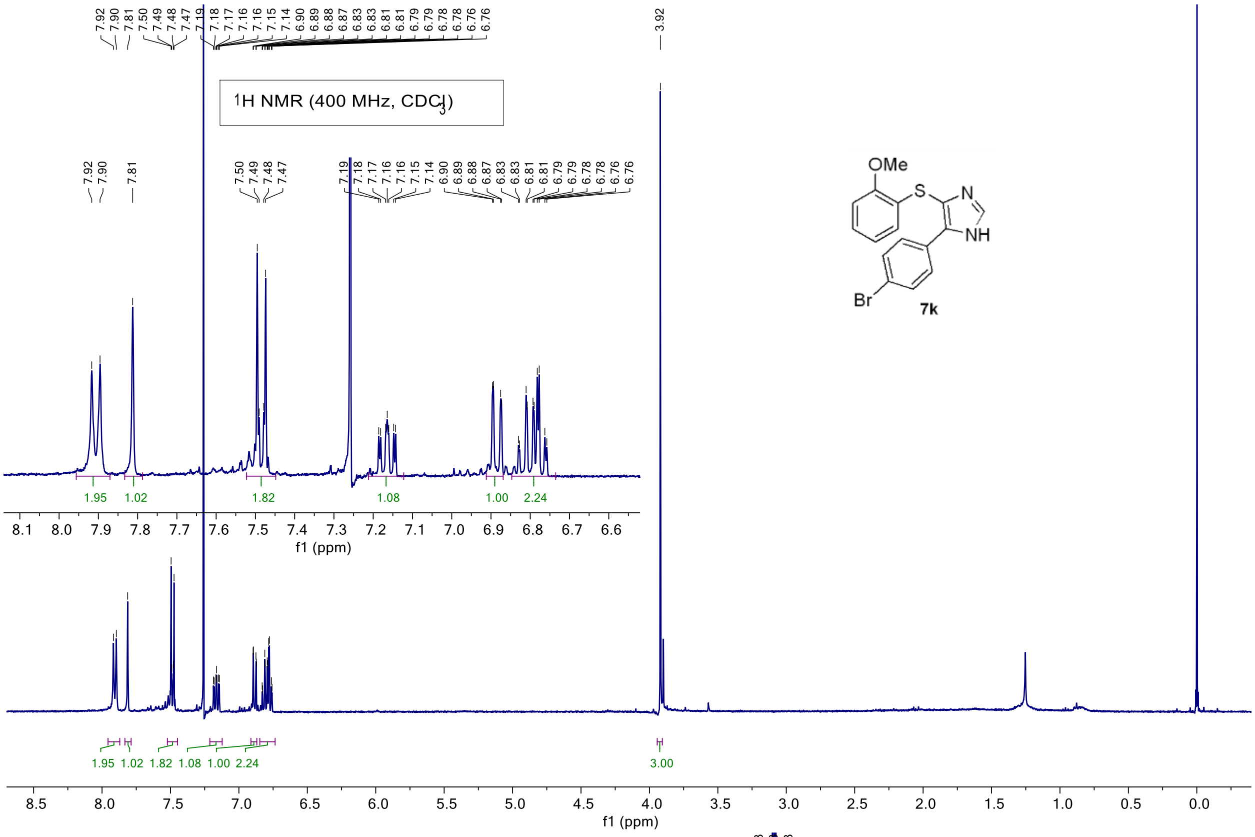

1H NMR (400 MHz, CDCl3)
OMe
S
N
NH
Br
7k
f1 (ppm)

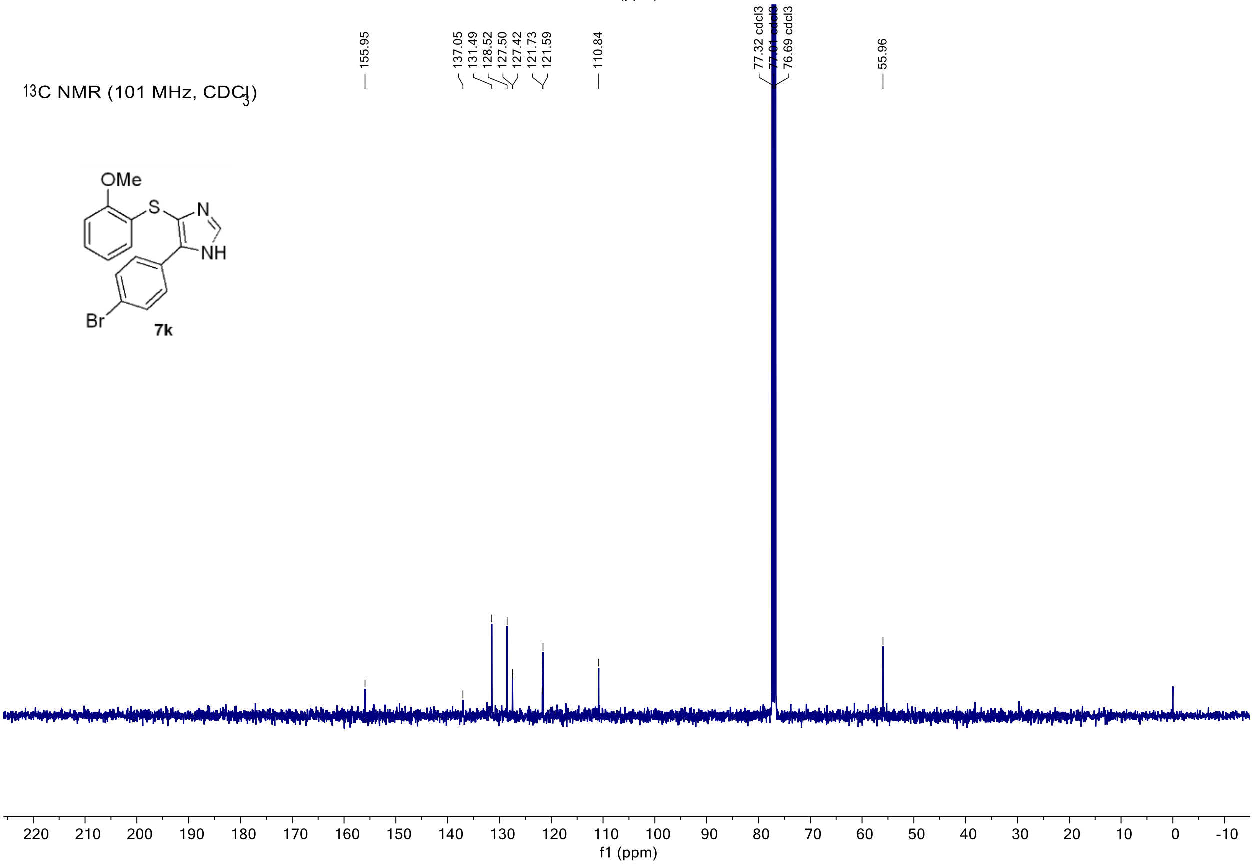

13C NMR (101 MHz, CDCl3)
OMe
S
N
NH
Br
7k
f1 (ppm)

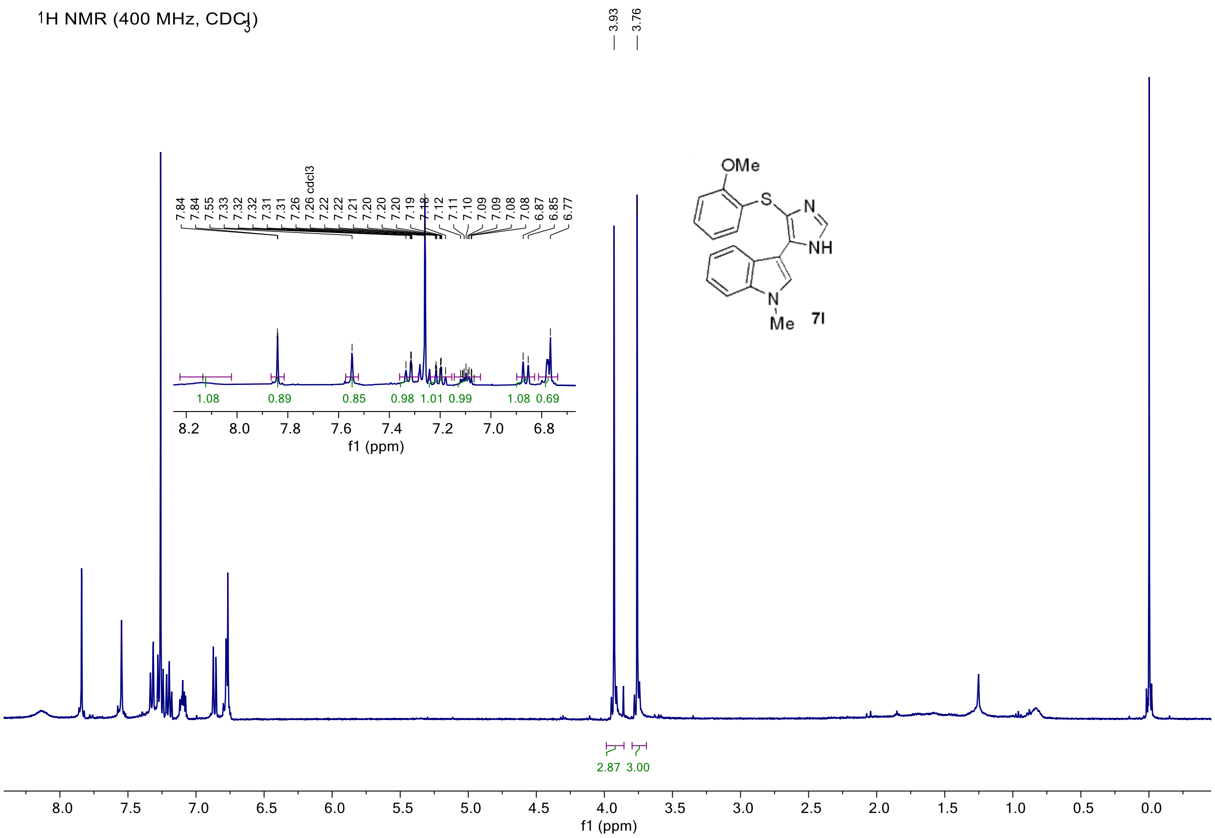
1H NMR (400 MHz, CDCl3)
3.93
3.76
7.84 7.84 7.55 7.33 7.32 7.32 7.31 7.31 7.26 7.26 cdcl3 7.22 7.22 7.21 7.20 7.20 7.20 7.19 7.18 7.12 7.11 7.10 7.09 7.09 7.08 7.08 6.87 6.85 6.77
OMe
S
N
NH
N
Me
7l
1.08 0.89 0.85 0.98 1.01 0.99 1.08 0.69
2.87 3.00
f1 (ppm)

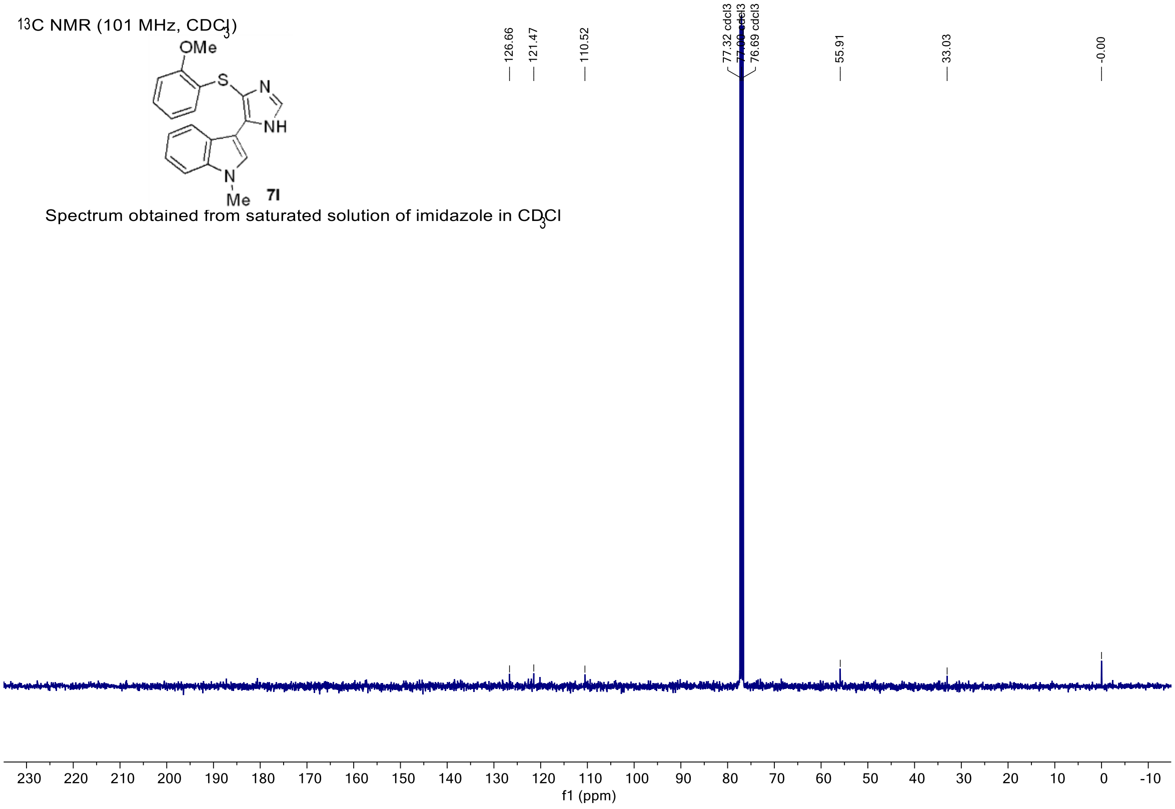
13C NMR (101 MHz, CDCl3)
126.66
121.47
110.52
77.32 cdcl3
77.00 cdcl3
76.69 cdcl3
55.91
33.03
-0.00
OMe
S
N
NH
N
Me
7l
Spectrum obtained from saturated solution of imidazole in CDCl3
f1 (ppm)

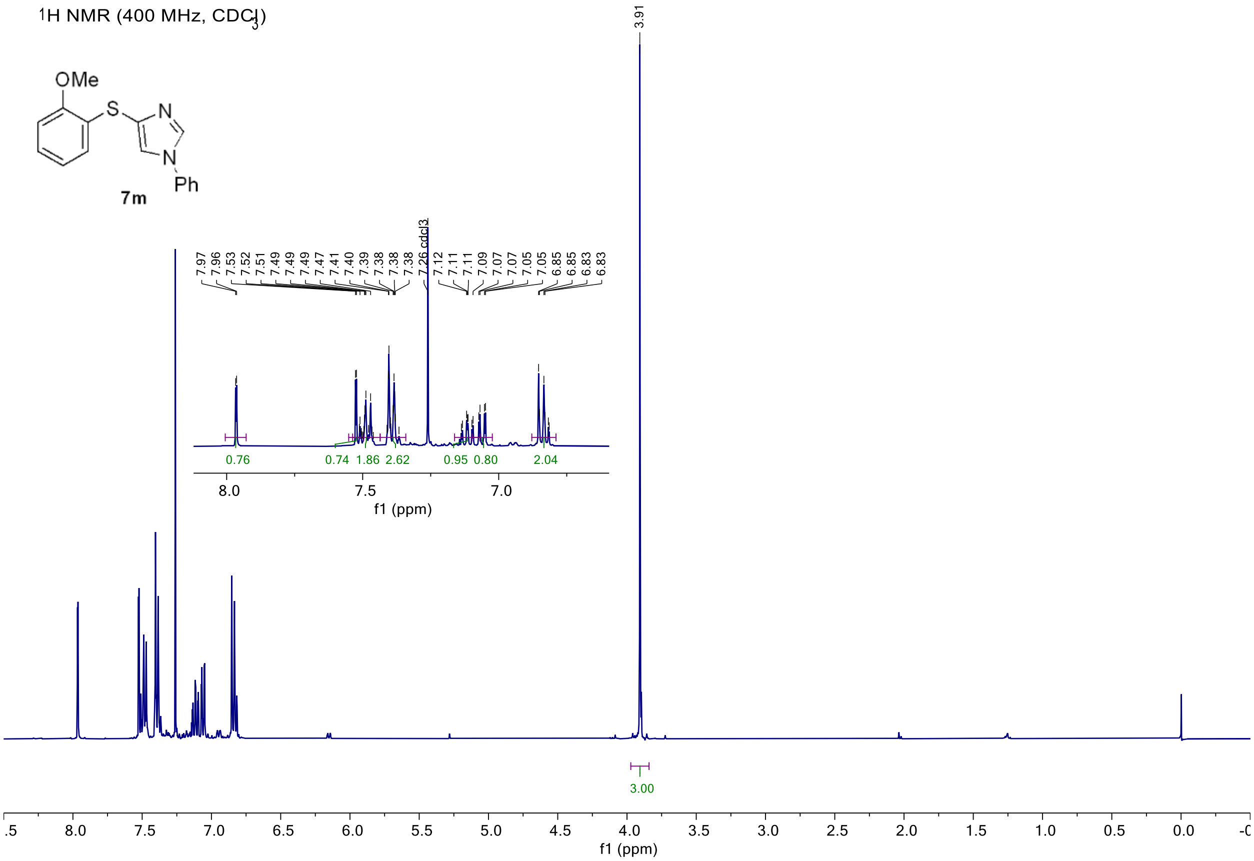

1H NMR (400 MHz, CDCl3)
OMe
S
N
N
Ph
7m
3.91
7.97
7.96
7.53
7.52
7.51
7.49
7.49
7.49
7.47
7.41
7.40
7.39
7.38
7.38
7.38
7.26 cdcl3
7.12
7.11
7.11
7.09
7.07
7.07
7.05
7.05
6.85
6.85
6.83
6.83
0.76
0.74
1.86
2.62
0.95
0.80
2.04
3.00
f1 (ppm)

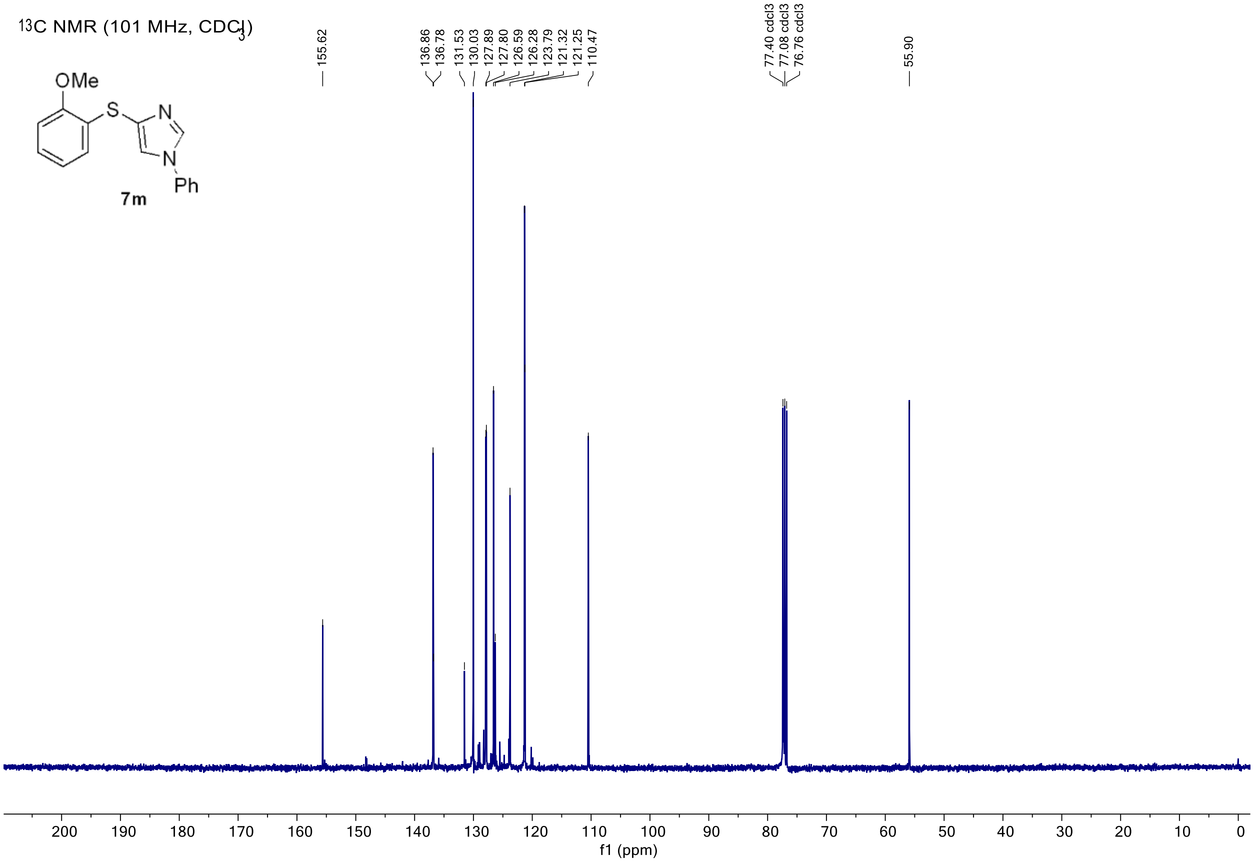

13C NMR (101 MHz, CDCl3)
OMe
S
N
N
Ph
7m
155.62
136.86
136.78
131.53
130.03
127.89
127.80
126.59
126.28
123.79
121.32
121.25
110.47
77.40 cdcl3
77.08 cdcl3
76.76 cdcl3
55.90
f1 (ppm)

1H NMR (400 MHz, CD_3OD)

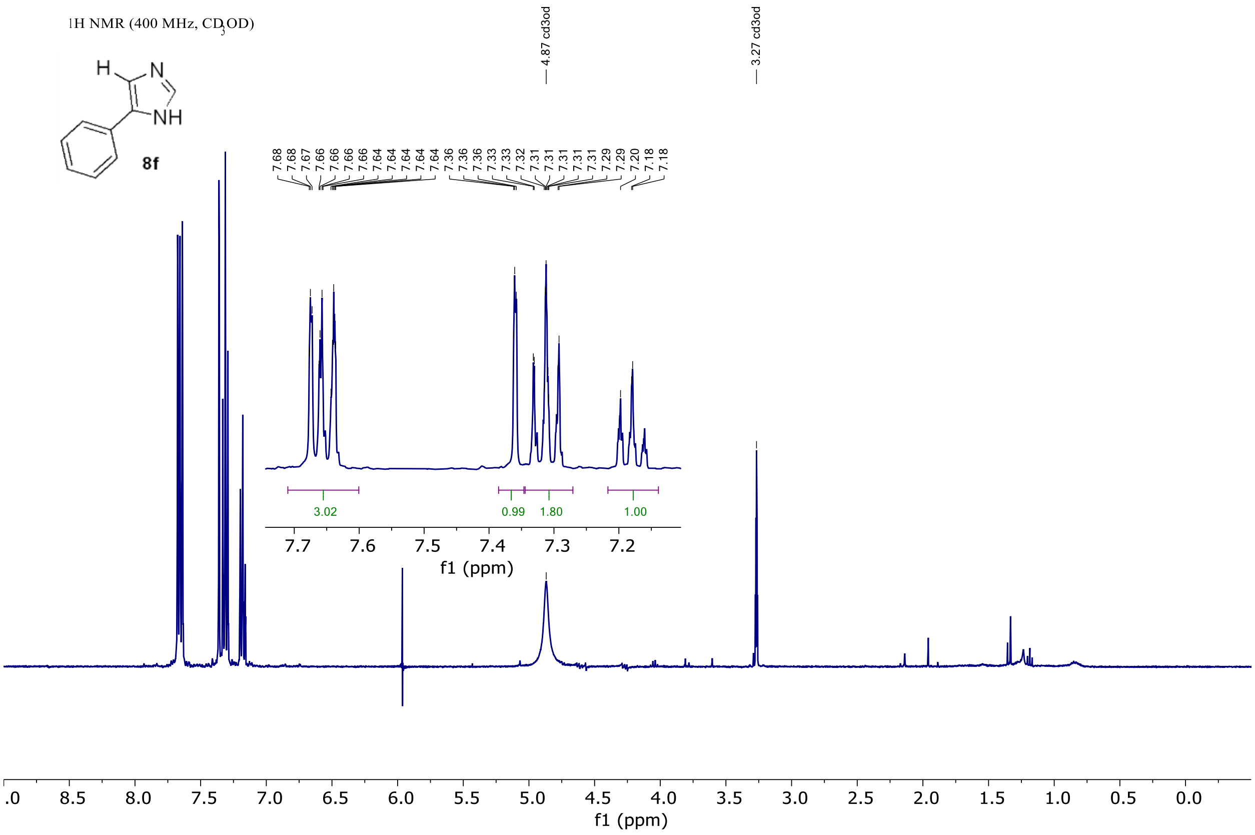

13C NMR (101 MHz, $CDCl_3$)

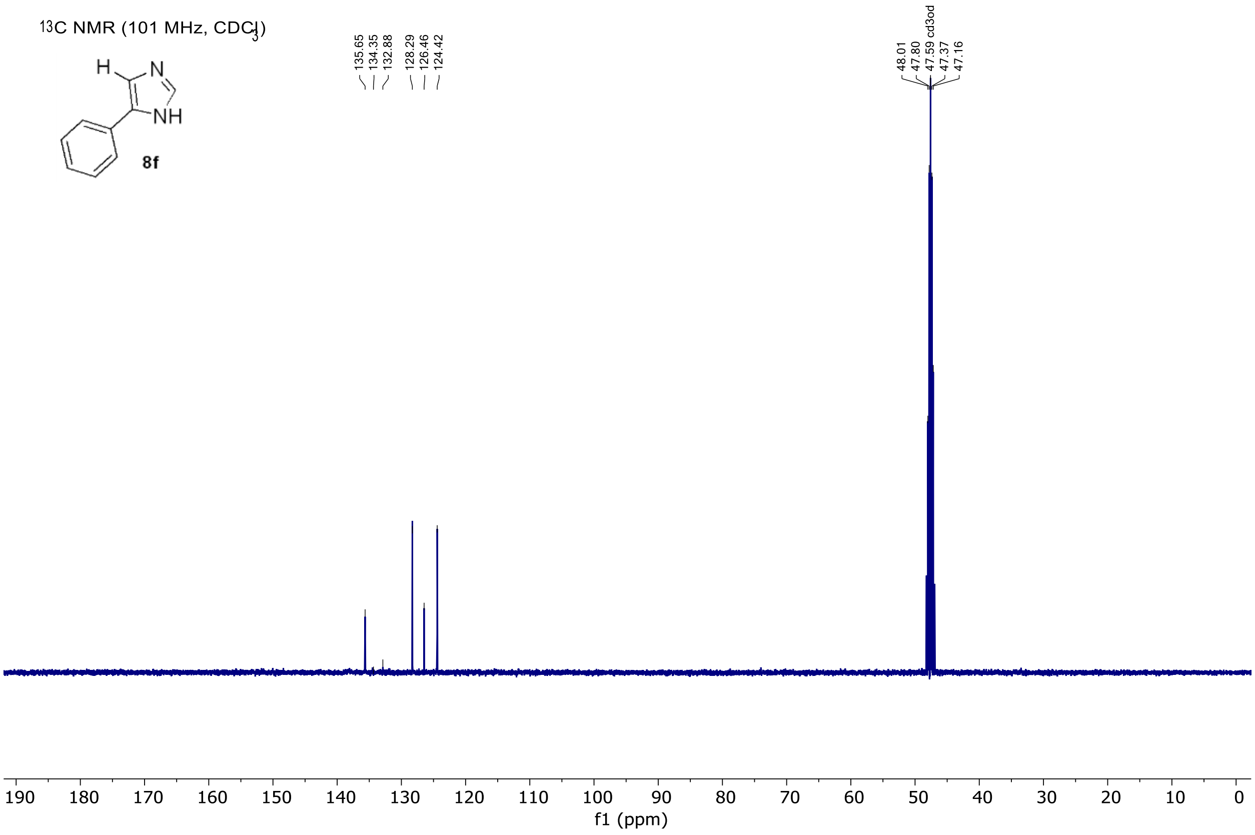

^{1}H NMR (400 MHz, $CDCl_3$)

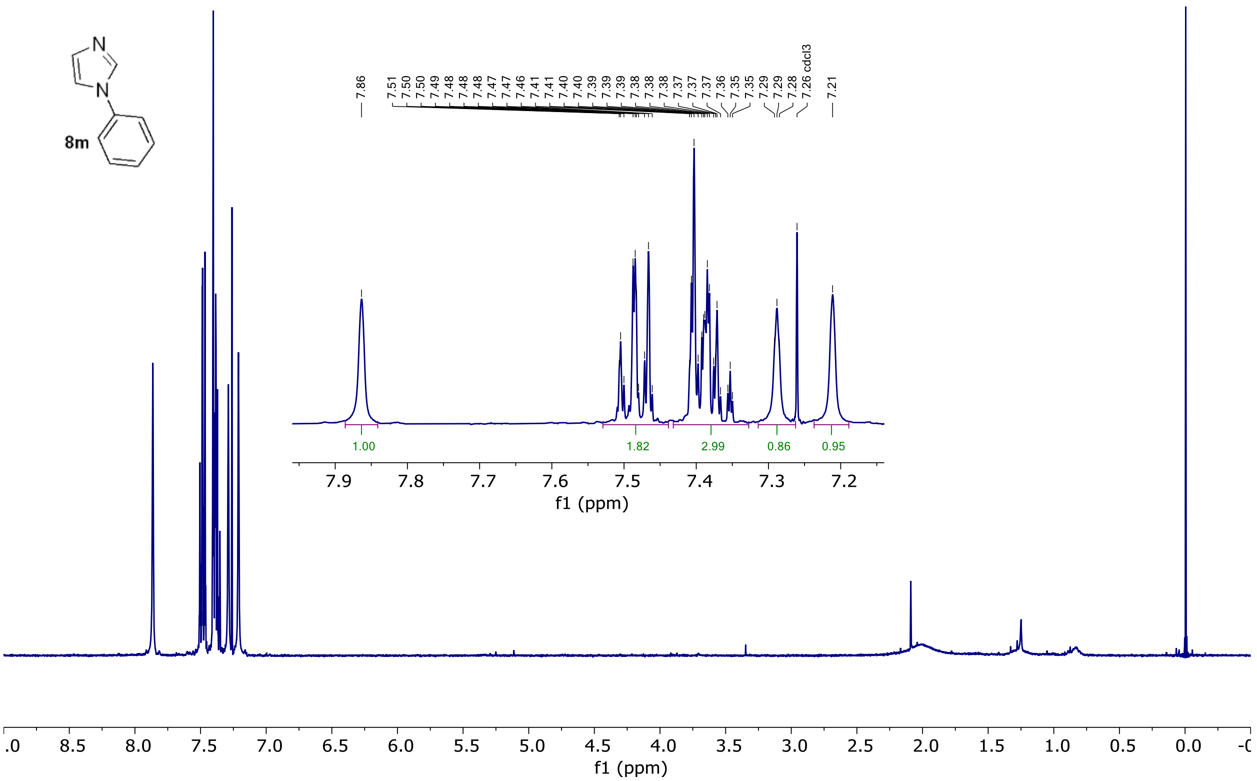

^{13}C NMR (101 MHz, $CDCl_3$)

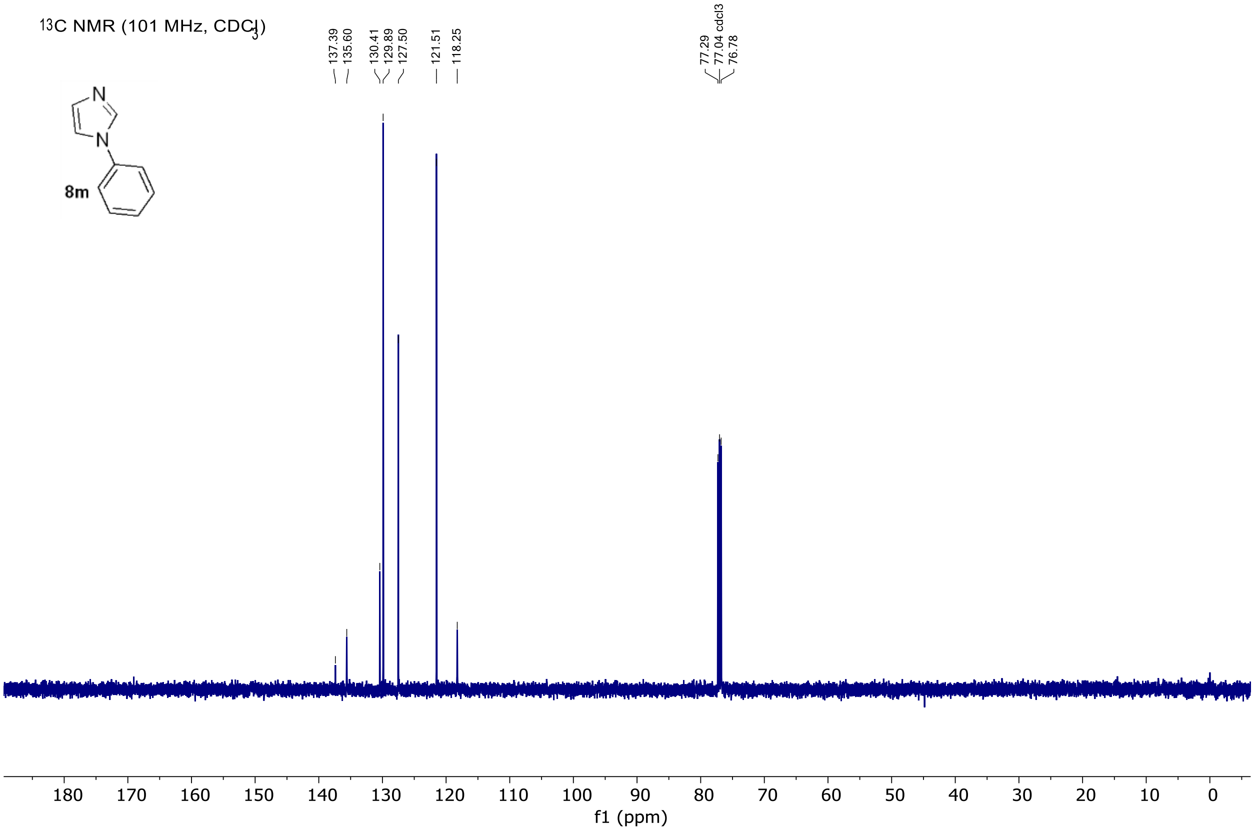

References

[1] Imidazoles lacking N-substituents have a low solubility in standard NMR solvents making quaternary carbon atoms difficult to detect by ^{13}C NMR (see: D. Pinto, C. M. Santos, A. M. Silva, *Recent Research Developments in Heterocyclic Chemistry*, **2007**, 397-475). All expected peaks appear in the ^{13}C spectrum of N-phenyl imidazole **7m** which was more soluble in $CDCl_3$ than the unsubstituted imidazoles. The best possible ^{13}C NMR spectra are provided given the solubility limitation.

[2] The 4 and 5 positions of imidazoles lacking N-substituents are often isochronous due to prototropic tautomerization (See: Nesmeyanov, A. N.; Zavelovich, E. B.; Babin, V. N.; Kochetkova, N. S.; Fedin, E. I. *Tetrahedron* **1975**, *31*, 1461-1462); imidazoles are drawn as the 4-((2-methoxyphenyl)thio) tautomer.

[3] Alwedi, E.; Lujan-Montelongo, J. A.; Pitta, B. R.; Chao, A.; Cortés-Mejía, R.; del Campo, J. M.; Fleming, F. F. *Org. Lett.* **2018**, *20*, 5910-5913.

[4] lmidazoles **7j-l** exhibited low solubility in CD_3OD, DMSO-d6 and acetone-d6 resulting in an inability to identify all the signals in the ^{13}C NMR.

[5] Bellina, F.; Cauteruccio, S.; Rossi, R. *J. Org. Chem.* **2007**, *72*, 8543-8546.

[6] Moghaddam, F. M.; Tavakoli, G.; Moafi, A.; Saberi, V.; Rezvani, H. R. *ChemCatChem.* **2014**, *6*, 3474-3481.
