# Supplementary material for: One-step synthesis of imidazoles from Asmic (anisylsulfanylmethyl isocyanide)
Source: Beilstein J Org Chem. 2021 Jun 24;17:1499–502. doi: 10.3762/bjoc.17.106 (PMC8239262; doi:10.3762/bjoc.17.106)
Supplement: File 2 — Raw FID files. [file Beilstein_J_Org_Chem-17-1499-s002.zip › FIDs for Publication/Read Me.pdf]

One-step Synthesis of Imidazoles from Asmic Isocyanide and Nitriles

Louis Mueller[a], Allen Chao,[b] Embarek Alwedi,[c] and Fraser Fleming*[a]

[a] Department of Chemistry. Drexel University 32 South 32nd Street Philadelphia, PA 19104, USA
[b] Wistar Institute, 3601 Spruce Street, Philadelphia, PA 19104
[c] Merck Inc., 90 E. Scott Ave, Rahway, NJ 07065.

FID data for the ^{1}H NMR and ^{13}C NMR spectra are available for the compounds in the table below. The files for each compound are in separate folders with subfolders titled 1H and 13C. MestreNova can be used to process the files. Spectra were collected on Varian Mercury Plus 400 (400 MHz/101 MHz) or Varian Unity Inova 500 (500 MHz/126 MHz) spectrometers at room temperature. Chemical shifts are reported relative to $CDCl_3$ (δ 7.26) for ^{1}H NMR and TMS (δ 0.00) for ^{13}C NMR.

Compound		
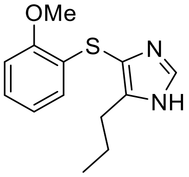 **7a**	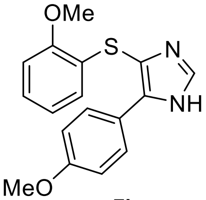 **7h**	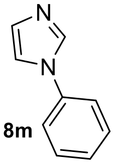 **8m**
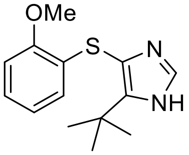 **7b**	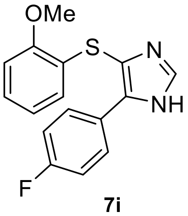 **7i**	
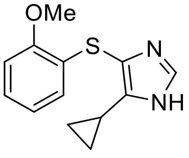 **7c**	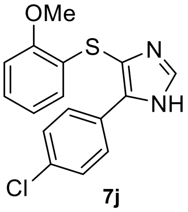 **7j**	
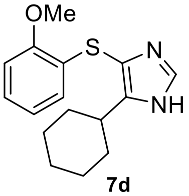 **7d**	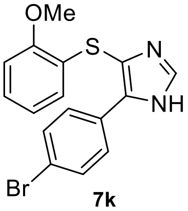 **7k**	

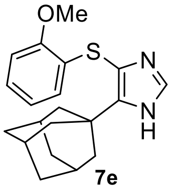

7e

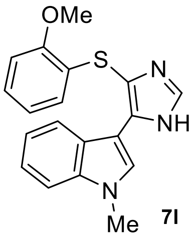

7l

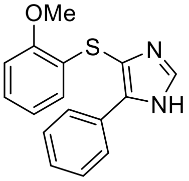

7f

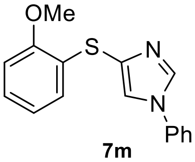

7m

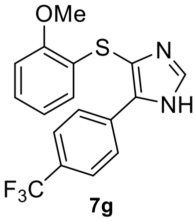

7g

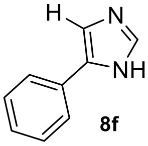

8f
